# Male meiosis in Crustacea: synapsis, recombination, epigenetics and fertility in *Daphnia magna*

**DOI:** 10.1007/s00412-015-0558-1

**Published:** 2015-12-21

**Authors:** Rocío Gómez, Kay Van Damme, Jaime Gosálvez, Eugenio Sánchez Morán, John K. Colbourne

**Affiliations:** 1 Departamento de Biología, Facultad de Ciencias, Universidad Autónoma de Madrid, E-28049 Madrid, Spain; 2 Environmental Genomics Group. School of Biosciences, University of Birmingham, Edgbaston, B15 2TT UK; 3 Chromosome Dynamics Group. School of Biosciences, University of Birmingham, Edgbaston, B15 2TT UK

**Keywords:** Meiosis, *Daphnia*, Synapsis, Recombination, Epigenomics, Fertility

## Abstract

We present the first detailed cytological study of male meiosis in *Daphnia* (Crustacea: Branchiopoda: Cladocera)—an aquatic microcrustacean with a cyclical parthenogenetic life cycle. Using immunostaining of the testes in *Daphnia magna* for baseline knowledge, we characterized the different stages of meiotic division and spermiogenesis in relation to the distribution of proteins involved in synapsis, early recombination events and sister chromatid cohesion. We also studied post-translational histone modifications in male spermatocytes, in relation to the dynamic chromatin progression of meiosis. Finally, we applied a DNA fragmentation test to measure sperm quality of *D. magna*, with respect to levels of inbreeding. As a proxy for fertility, this technique may be used to assess the reproductive health of a sentinel species of aquatic ecosystems. *Daphnia* proves to be a model species for comparative studies of meiosis that is poised to improve our understanding of the cytological basis of sexual and asexual reproduction.

**Electronic supplementary material:**

The online version of this article (doi:10.1007/s00412-015-0558-1) contains supplementary material, which is available to authorized users.

## Introduction

Meiosis is the most fundamental biological process that is shared among sexually reproducing eukaryotes. Because of its early evolutionary origin, its importance for reproduction and its impact on fitness, the mechanisms and components of meiosis are highly conserved. Meiosis is a specialized cell division program that consists of two consecutive rounds of cell division, following a single round of DNA replication, thereby forming haploid gametes from diploid germ cells. Failures during this process lead to errors in chromosome segregation, which are a major cause for miscarriages and birth defects in animals. To ensure the correct transmission of chromosomes during both meiotic divisions, chromosome sister chromatids must be held together by the Cohesin Complexes through an event called sister chromatid cohesion (SCC), which must be tightly regulated for chromosomes to properly segregate. During synapsis, homologous chromosomes must first recognize each other, pair, then closely associate by a proteinaceous structure called the synaptonemal complex (SC). Recombination events introduce genetic variability and mediate the pairing of homologous chromosomes during prophase, which ultimately ensures the accurate segregation of homologous chromosomes during the first meiotic division. Much of this knowledge about meiosis originates from studies in model species such as yeast (Kerr et al. 2012), *Drosophila melanogaster* (Lake and Hawley 2013), *Caenorhabditis elegans* (Rog and Dernburg 2013), *Mus musculus* (Bolcun-Filas and Schimenti 2012) and *Xenopus laevis* (Philpott and Yew 2008), yet also from *Arabidopsis thaliana* as the icon of plant biology (Tiang et al. 2012). Although meiosis is conserved across animals, there are substantial differences in how it is regulated among different species. And although the study of meiosis has progressed rapidly over the past 15 years (Keeney 2009) by providing a better understanding of the genes and proteins involved, there are still gaps in knowledge about the mechanisms of meiosis in large taxonomic groups such as the Crustacea, thereby making detailed comparative studies with other major invertebrate lineages difficult.

Crustaceans consist of over 67,000 described species forming a distinct lineage among the Arthropoda. The diversity in their types of reproduction is of significant scientific value. For example, the cladocerans (or water fleas) consist of over 620 described species (Forró et al. 2008) that show a cyclical parthenogenetic reproductive strategy, where asexual reproduction (parthenogenesis) occasionally alternates with sexual reproduction, which creates diapausing embryos after the fertilization of haploid gametes obtained via female and male meiosis. This switch from asexual to sexual reproduction in response to environmental cues allows populations to more rapidly adapt to harsh conditions because of greater genetic diversity through meiotic recombination. Consequently, meiosis is the key event to be investigated so to understand how production of haploid oocytes and spermatozoa occur, and what molecular and cytological mechanisms allow gametes to accomplish successful fertilization. The majority of studies about meiosis conducted so far using obligate sexual crustaceans have used classical histological techniques, or orcein staining, and have rarely used electron microscopy. Studies using male marine shrimps *Fenneropenaeus chinensis* (Xie et al. 2008) and *Litopenaeus vannamei* (Garza-Torres et al. 2011; Heitzmann et al. 1993) concluded that spermatogenesis is a continuous process, unrelated to the crustacean moult cycle. Cytogenetic studies using male brine shrimps *Artemia franciscana* (Papeschi et al. 2000) and *A. persimili* (Rodriguez-Gil et al. 1998) were limited to describing the first stages of male meiosis (no stages further than diakinesis). Although these studies are helpful introductions to meiosis in Crustacea, none have yet shown the complete staging of meiosis in males, nor any specific distribution of proteins implicated in meiosis progression.

We chose to advance knowledge of meiosis in Crustacea by studying the cyclical parthenogenetic model species *Daphnia* (Crustacea: Branchiopoda: Cladocera), which are microcrustaceans and keystone elements of freshwater ecosystems. As a model organism, *Daphnia* species possess the necessary characteristics that permit experiments under reproducible and uniform research conditions (Lampert 2011)*. Daphnia* is an important organism in ecological, evolutionary and environmental genomics (Colbourne et al. 2011), epigenetics (Harris et al. 2012) and ecotoxicological genomics (Shaw et al. 2008) research, including life history studies of the transition from asexual to sexual reproduction (Zaffagnini 1987). The *Daphnia* phylogeny indicates a single shift in chromosome numbers during the evolution of the genus; species like *D. pulex* have 12 pairs of chromosomes (2*n* = 24) whereas species like *Daphnia magna* have 10 pairs of chromosomes (2*n* = 20)—the latter number being ancestral (Beaton and Hebert 1994; Colbourne et al. 1997). The sequencing of *D. pulex* genome is accelerating the pace of discoveries (Colbourne et al. 2011). The expression patterns of meiosis-related and meiosis-specific genes in female *D. pulex* was described (Schurko et al. 2009), and data on the genes associated for meiosis inactivation in obligate asexual lineages were reported (Eads et al. 2012; Lynch et al. 2008; Xu et al. 2013). Although the cyclical reproductive strategy of *Daphnia* is well known for decades (Zaffagnini and Sabelli 1972), the molecular and cytological mechanisms governing their modes of reproduction are still unknown and are certainly worth exploring. Yet, the size of the animals and of their chromosomes has slowed cytological investigations on *Daphnia* meiosis*. Daphnia* adults are typically 1–3 mm in length, thus tissues and especially the small chromosome sizes are the major impediments to the cytological examination of *Daphnia*’s meiocytes; karyological observations revealed that the first and largest chromosome measured 5.6–6.6 μm or 25 % of the total (Colbourne et al. 2011). Partial cytological observations of mitotic and meiotic chromosomes were made in *D. pulex* using classical methods such as hematoxilin/eosin or Giemsa staining, and the aceto-orcein squash method (MacQueen et al. 2005; Ojima 1958; Staiber 2012; Zaffagnini and Sabelli 1972). Recently, *Daphnia* meiotic cytology (in *D. pulex*) was significantly advanced by the publication of specific methods for meiotic chromosome preparation, immunofluorescence and fluorescence in situ hybridization (Tsuchiya et al. 2009).

Following these earlier works, we pursued four avenues for our own study, focusing on *D. magna* meiosis in males:

### First

we identified all male meiotic stages and spermiogenesis, since the complete process was still uncharacterized.

### Second

we described the temporal and functional relationships between DNA events in early meiotic recombination and synapsis, by analysing the distribution of the γH2AX marker, since it labels the Double Stranded Breaks (DSBs) during early meiotic recombination events (Rogakou et al. 1998). We also used recombinase Rad51 as a marker of later recombination events, which is known to be recruited to DSBs downstream through the recombination pathway (Barlow et al. 1997). Synapsis was also studied by analysing the distribution of SMC3, which is a cohesin complex subunit that is widely conserved in eukaryotes (Hirano 2002). SMC3 is a member of the Structural Maintenance of Chromosomes (SMC) family and is commonly used because of its location along the cohesin axes underlying the axial elements (AEs) and lateral elements (LEs) of the SC in several organisms such as *Mus musculus* (Pelttari et al. 2001), *Rattus norvegicus* (Eijpe et al. 2003). *Locusta migratoria (Viera et al.*^2004^*)*, *Stethophyma grossum* (Calvente et al. 2005), *Caenorhabditis elegans* (Mito et al. 2003) and *Arabidopsis thaliana* (Lam et al. 2005) meiosis.

### Third

we investigated the *Daphnia* epigenetic repertoire during male meiosis. *Daphnia* are particularly good models for epigenetic studies, because their cyclical parthenogenetic reproductive cycle allows the study of epigenetic differences across conditions and treatments in the absence of genetic variation. Differences between the sexes are likely epigenetically determined, since males are genetically identical to their mothers and sisters (Zaffagnini 1987) and sex is determined by environmental cues (Strahl and Allis 2000). There is growing consensus that histone regulation controls DNA accessibility (Bannister and Kouzarides 2011). The four core histones are subject to over a hundred different histone post-translational modifications that are believed to precisely regulate the chromatin structure and function (Kouzarides 2007). We investigated the epigenetic patterns of *Daphnia,* which includes modified histones (Robichaud et al. 2012), and observed cytological expression of epigenetic protein modifications in *Daphnia* males, during both meiotic divisions.

### Fourth

we pursued a novel method to study cladoceran male fertility by quantifying the quality of *Daphnia* sperm. Males are produced from diploid asexual eggs under environmental control (e.g., diminishing resources) (Hebert 1978). These mature males mate with receptive females that produce a limited number of haploid eggs. After fertilization, an embryo is encased in a protective shell (ephippium) and can persist in a diapause state until favourable conditions return (Grebelnyi 1996). For the successful fertilization and offspring hatch, male fertility is an essential determinant that regulates population survival.

Studies focused on *Daphnia* reproduction are insightful for understanding the mechanisms of sexual and asexual reproduction in animals, yet they also provide a link between genetic variability, cytological expression and environmentally determined reproduction strategies. This study is a starting point for this line of research.

## Material and methods

### *D. magna* isolates and culturing

We used *D. magna* to describe *Daphnia* male meiosis. Two distinct ecotypes were selected. The first is an isoclonal isolate called UoB1, which was sampled from a natural population in June 2013 at Edgbaston Pool, Birmingham, West Midlands, UK (coordinates 52°27′12.9″N 1°55′10.0″W), a UK SSI (Site of Specific Interest). The second ecotype is derived from the X isoclonal line collected by Prof D. Ebert (University of Basel) from a rock pool on a Skerry island near Tvärminne, Finland (59.833183, 23.260387) (Routtu et al. 2010). We also used Xinb1 and Xinb3 isolates, which are the first and third inbred descendants of the X isolate, respectively (Routtu et al. 2010). Xinb3 is the isolate used for the ongoing *D. magna* genome project. All experiments for our study was done using *natural males*, meaning males produced in response to a combination of crowding and reduced food quantity in each of the correspondent isolates (Olmstead and LeBlanc 2001).

All *D. magna* cultures were kept at optimal temperature conditions (20 °C ± 2 °C) in 2 L beakers containing 1200 ml of HHCOMBO medium according to the original protocol (Baer and Goulden 1998), and modified by addition of 0.002 mg mL^−1^ sodium selenite (OECD 1998; OECD 2004) (Keating and Dagbusan 1984). Cultures were maintained with a light:dark photoperiod of 16:8 h. The medium was changed weekly, and animals were fed three times weekly a concentrated monoculture of unicellular green algae (*Chlorella vulgaris*). Algae were grown under a constant source of photosynthetic light and aeration at 20 °C in Bold’s basal medium (BBM).

### Spermatocyte preparations

Adult males were fixed, then the testes were dissected and processed based on the squashing procedure previously described (Page et al. 1998) and specifically modified for *Daphnia* (Tsuchiya et al. 2009). Briefly, animals were fixed in freshly prepared 2 % formaldehyde in PBS (137 mM NaCl, 2.7 mM KCl, 10.1 mM Na_2_HPO_4_, 1.7 mM KH_2_PO_4_, pH 7.4) containing 0.05 % Triton X-100 (Sigma). Testes were rapidly dissected with the animal immersed in the fixation solution with tungsten dissection needles under a stereomicroscope. After dissection, testes were left immersed in the fixation solution for an additional 5 min. Testes were then individually placed onto a slide coated with 1 mg/ml poly-l-lysine (Sigma) with a small drop of fixative, and gently minced with tweezers. The testes were then squashed and the cover slip removed after freezing in liquid nitrogen (−80 °C).

### Immunofluorescence microscopy

For the immunolabelling technique, we followed protocols described for mammalian spermatocytes (Page et al. 1998) as follows. After fixation, the squashed spermatocyte preparations were rinsed three times for 5 min in PBS, and incubated overnight at 4 °C with the corresponding primary antibodies diluted in PBS. In double-labelling experiments, primary antibodies from different host species were incubated simultaneously. Following three washes in PBS for 5 min, the slides were incubated for 30 min at room temperature with the corresponding secondary antibodies. The slides were subsequently rinsed in PBS and counterstained for 3 min with 10 μg/ml DAPI (4′,6-diamidino-2-phenylindole). After a final rinse in PBS, the slides were mounted with antifading mounting media Vectashield (Vector Laboratories) and sealed with rapidly solidifying nail varnish. Preparations were kept at 4 °C until observation.

Immunofluorescence image stacks were collected using a NIKON Eclipse 90i microscope equipped with epifluorescence optics, a motorized z-drive, and a Hamamatsu ORCA-ER C4742-80 digital camera controlled by Nis-Elements AR Software. Stacks were analysed and processed using the NiS-Elements^TM^ software or the public domain ImageJ^TM^ software (National Institutes of Health, USA; http://rsb.info.nih.gov/ij). 3D reconstructions for supplementary videos were also made with NIKON software. Final images were edited using Adobe Photoshop software.

### Antibodies

For immunofluorescence staining, the following primary antibodies were used at the indicated dilution: rabbit serum K987 against human SMC3 (Prieto et al. 2004), kindly provided by Dr. Barbero (CIB/CSIC, Spain) at 1:30; human anti-centromere autoantibody (ACA serum) revealing kinetochores (Antibodies Incorporated, 15-235) at 1:30; monoclonal mouse antibody against γH2AX (Millipore, 05-636) at 1:500; rabbit polyclonal antibody Rad51 (Calbiochem PC130) at 1:30; rabbit polyclonal antibody against H2AT120ph (Active Motif, 3939) at 1:100, rabbit polyclonal antibody against H3S10ph (Millipore, 06-570) at 1:70, rabbit polyclonal antibody against H3K9me3 (Abcam, ab8898) at 1:100; and monoclonal FITC Labelled antibody against αTubulin (Sigma, F2168) at 1:150. The secondary antibodies used were: donkey anti-rabbit IgG (Jackson) and donkey anti-mouse IgG (Jackson). All antibodies were employed at a 1:150 dilution in PBS, and were conjugated with either Texas Red or fluorescein isothiocyanate (FITC).

### Western blot

To determine the specific immunoreactivity of anti-SMC3, anti-γH2AX, anti-Rad51, anti H3S10ph, anti-H3K9m3 and anti-αTubulin polyclonal antibodies, we performed Western Blot analysis of *Daphnia* testes extracts. *Daphnia* testes were removed and disaggregated in RIPA buffer according to previous Western Blot protocols in this species (Pijanowska and Kloc 2004). The blot was incubated with the correspondent antibodies at a dilution of 1:200 each, followed by incubation with HRP-conjugated donkey anti-rabbit or anti-mouse IgG at a dilution of 1:2000 (Amersham). Visualization was performed using alkaline phosphatase detection system (BioRad).

### TUNEL assay

The DNA fragmentation-associated apoptosis of spermatocytes from inbred Xinb3 and UoB1 isolates was detected by the TdT-mediated dUTP-fluorescein nick end labelling (TUNEL) assay, using a commercial product (Roche, 11684795910). Nuclei were counterstained for 3 min with 10 μg/ml DAPI. The experiments on testes were conducted using formaldehyde-fixed squashed spermatocytes of natural Xinb3 and UoB1 males.

### Sperm quality assays

#### Sperm chromatin dispersion assay

Quantification of DNA fragmentation in *D. magna* sperm was conducted following the instructions of the Sperm DNA Fragmentation Test from Halosperm® (Halotech DNA SL, ISO 13485), which is based on Sperm Chromatin Dispersion. Sperm from *D. magna* males from UoB1, Xinb1 and Xinb3 isolates was obtained from live animals spontaneously ejaculating upon slowly opening the carapace of the animal with tungsten dissection needles under a stereomicroscope, when immersed in the original medium of culture. The sperm was immediately prepared to proceed with the DNA damage assay. Approximately 25 μl of diluted spermatozoa were added to an eppendorf tube with low melting point agarose at 37 °C and mixed thoroughly. Approximately 10–20 μl of the sperm suspension was spread onto a pre-treated microgel slide provided in the Halosperm ® kit, covered with a coverslip and cooled at 4 °C for 5 min on a pre-chilled surface. The coverslip was then carefully removed, and the slide placed horizontally. One drop of the lysing solution provided within the kit was added to the material. Finally, the slides were washed in distilled water for 5 min and dehydrated in a sequential series of ethanol baths (70 % and 100 % *v*/*v*) and then air-dried. Slides were stained with 10 μg/ml DAPI.

#### In situ nick translation

To validate the results of the Sperm Chromatin Dispersion assay, In Situ Nick Translation of the DNA breaks was performed on sperm samples treated with the lysing agent provided in the Halosperm® kit. After protein lysis of embedded spermatozoa in agarose microgel, the slides were thoroughly washed four times in PBS for 5 min each and then incubated for 5 min in an excess of reaction buffer for DNA polymerase I (10 mM Tris–HCl, 5 mM MgCl_2_, 7.5 mM DTT, pH 7.5). Following this treatment, 100 ml of reaction buffer containing 25 units of DNA polymerase I (New England BioLabs, Beverly, USA) and biotin-16-dUTP (Roche, Spain) in the nucleotide mix, was deposited onto the slide, covered with a plastic coverslip and incubated in a moist chamber for 25 min at 37 °C. After washing in TBE buffer (Sigma, St Louis, MO, USA), the slides were dehydrated in sequential series of ethanol baths (70, 90, and 100 % *v*/*v*) and air-dried. The incorporated biotin-16-dUTP was detected with an appropriate antibody conjugated with FITC (Roche, Spain) for 30 min. Slide preparations were counterstained with propidium iodide (2 μg/ml) and mounted with Vectashield.

## Results

### Immunoblotting

To corroborate the specificity of the antibodies used in the study, we performed Western Blot analyses with extracts of *D. magna* testes. For each of the antibodies against SMC3, γH2AX, Rad51, H3S10ph, H3K9m3 and αTubulin, a specific band was recognized corresponding to the expected molecular weight based on sequence homology and protein size (supplementary Fig. 1).

### Description of the stages in *D. magna* male meiosis

**Fig. 1:**
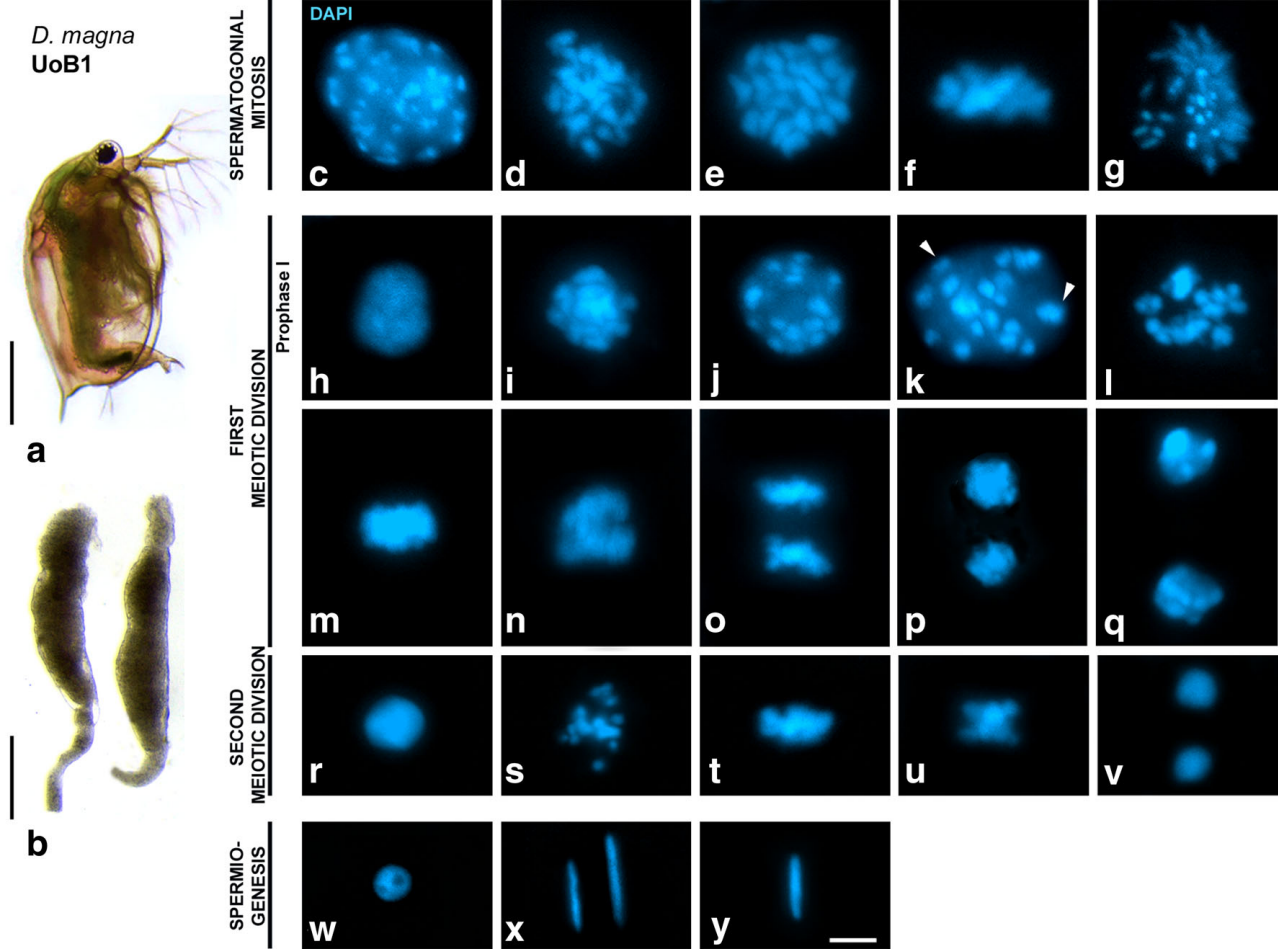
*Daphnia magna* male meiosis stages in UoB1 line. Counterstaining of the chromatin with DAPI (*blue*). **a** *Daphnia magna* male, *scale bar* corresponds to 1 mm. **b** Testis. *Scale bar* corresponds to 0.2 mm. **(c-g)** Spermatogonial mitosis: **c** prophase, **d** prometaphase, **e** polar metaphase, **f** lateral metaphase, **g** anaphase. **(h–q)** First meiotic division. **h** leptotene, **i** pachytene, **j** diplotene, **k** diakinesis, *white arrowheads* point chromocenters, **l** prometaphase I, **m** metaphase I, **n** early anaphase I, **o** late anaphase I, **p** early telophase I, **q** late telophase I. **(r-v)** Second meiotic division. **r** interkinesis, **s** prometaphase II, **t** metaphase II, **u** early anaphase II, **v** telophase II. **(w-y)** Spermiogenesis. **w** early spermatid, **x** medium spermatid, **y** mature spermatid. All images are projections of different focal planes throughout the cell volume. *Scale bar* in **y** corresponds to 2.5 μm. Detailed descriptions of the figure are located in Supplemental Figure Legend 1

**Fig. 2:**
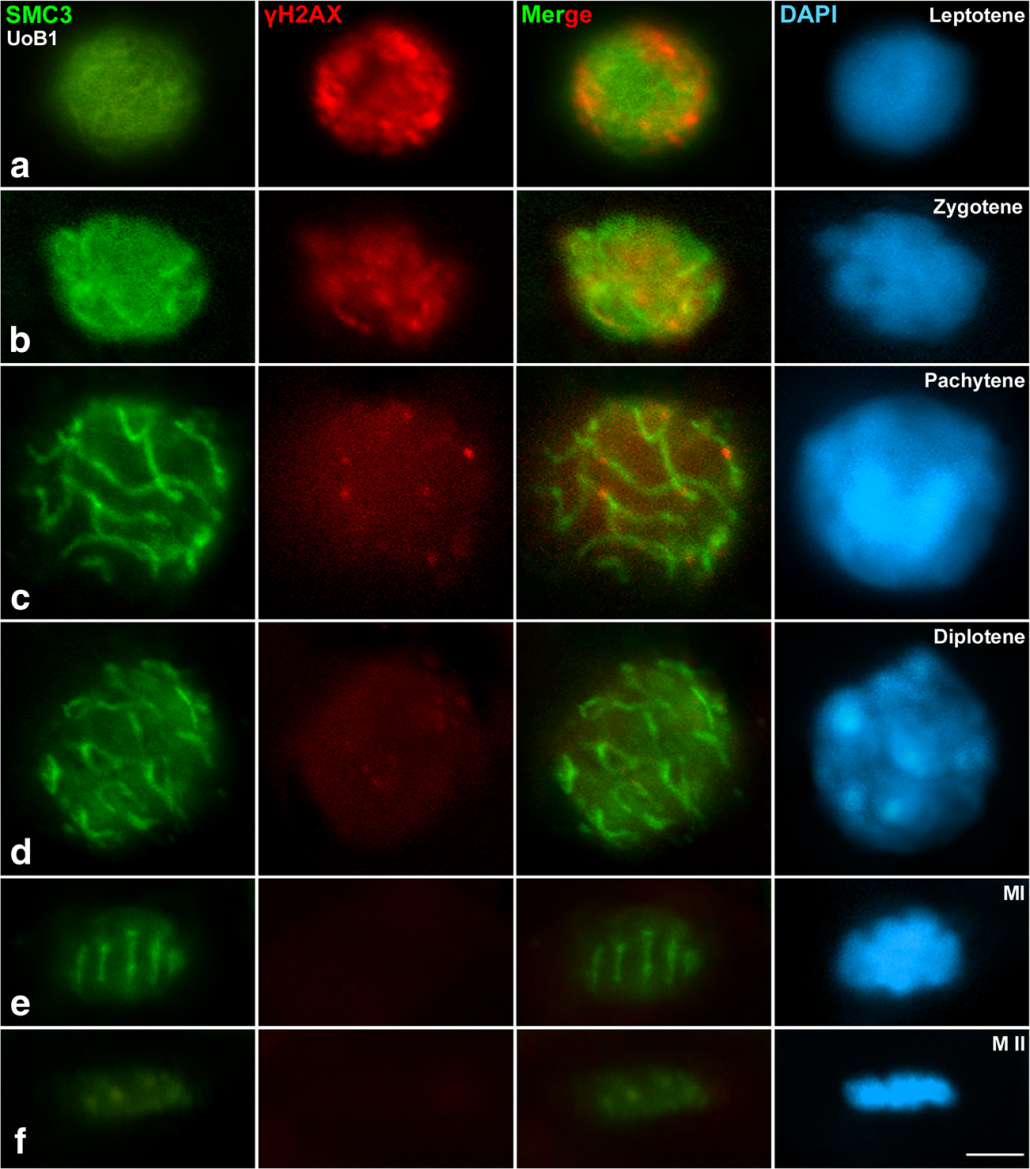
The progression of synapsis and initiation of recombination. Double immunolabelling of SMC3 (*green*) as marker for synapsis progression, and γH2AX (*red*) as marker for early recombination events. Counterstaining of the chromatin with DAPI (*blue*). The initiation of recombination, as detected by formation of DSBs (labelled by γH2AX), occurs before the initiation of synapsis. **a** leptotene, **b** zygotene, **c** pachytene, **d** diplotene, **e** metaphase I, **f** metaphase II. Additional information for this legend is offered in the online supplementay material. *Scale bar* in **f** corresponds to 2.5 μm

**Fig. 3:**
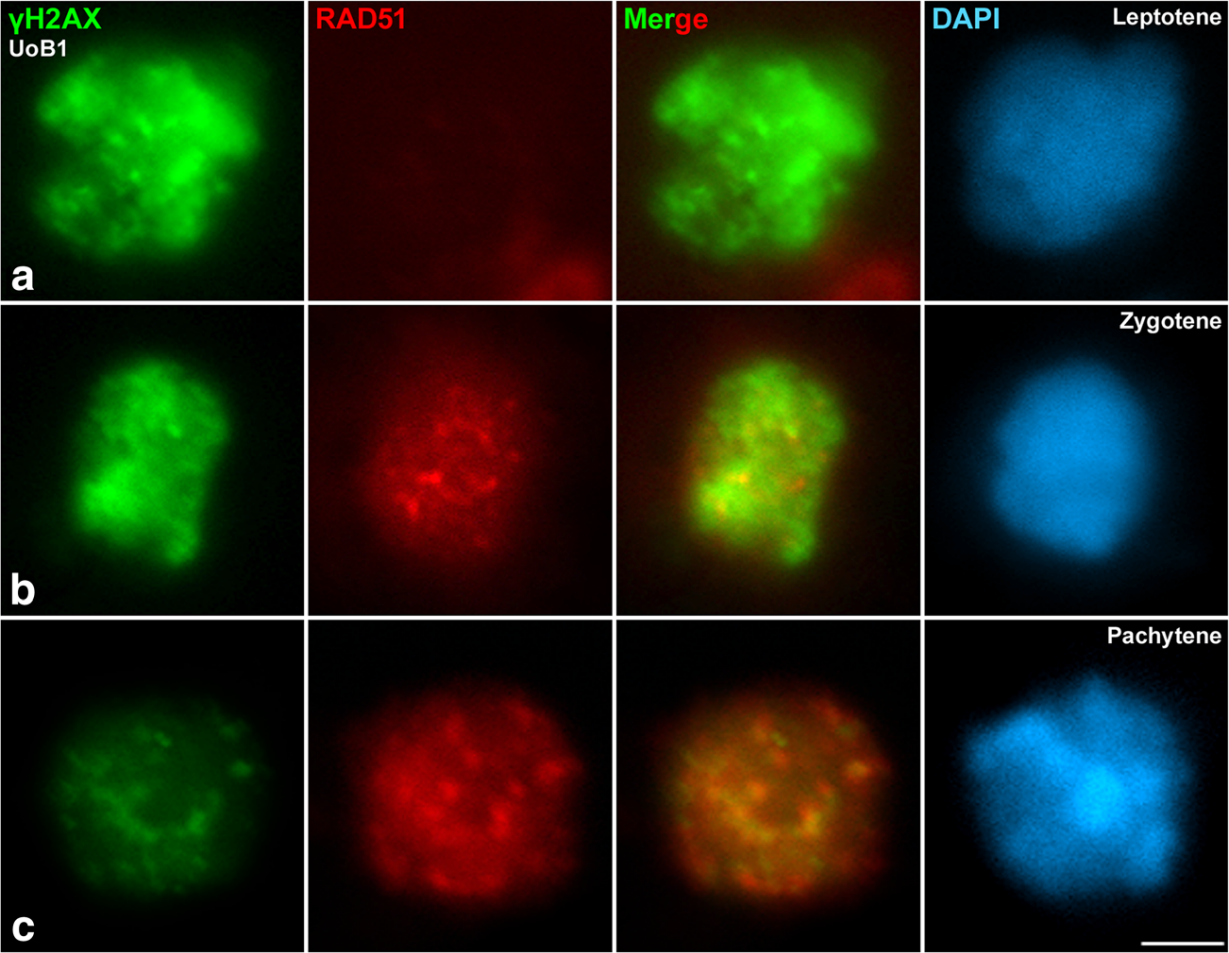
The progression of γH2AX and Rad51 from leptotene to pachytene. Double immunolabelling of γH2AX (*green*) as marker for formation of DSBs, and RAD51 (*red*) as marker for interhomologue recombination events. Counterstaining of the chromatin with DAPI (*blue*). Rad51 foci appear downstream of DSB formation immediately after γH2AX. **a** leptotene, **b** zygotene, **c** pachytene. *Scale bar* in **f** corresponds to 2.5 μm

A first essential step for our investigation was to define spermatogonias, meiotic stages and spermiogenesis in *D. magna*. We therefore stained squashed testis from *D. magna* UoB1 natural males (Fig. 1a, b) with DAPI, to identify cells according to morphology and chromatin condensation (Fig. 1). We specifically used the squashing technique so to not disturb chromosome condensation and distribution in dividing spermatocytes, and to also allow the proper differentiation of all meiotic stages (Page et al. 1998). With no previously detailed characterization of male meiosis in a crustacean, we first delineated the stages as a reference for this and future studies. It is important noting that these stages were strictly correlated to the results obtained with immunolocalization of axial elements (AEs) and lateral elements (LEs) of the synaptonemal complex (SC), and with specific markers for early recombination events (γH2AX and RAD51). These immunostaining meiotic stages are presented in Figs. 2 and 3 yet, in order to provide a guide for future studies, we first described the cytological chromatin appearance of the meiotic stages. All stages of spermatogonial mitosis and all stages of meiosis (both first and second meiotic division, together with stages of spermiogenesis) are shown in Fig. 1. Additional detailed descriptions of the stages are offered in the supplementary material.

To understand the progression of spermatogenesis in *D. magna*, we subsequently dissected an entire testis, which was stained with DAPI, and serially imaged to reconstruct the entire organ. Gonads in *D. magna* males consist of two testes. Each testis is a tubule of approximately 1 mm in length (Fig. 1b and Supplementary Fig. 2), and meiosis progress throughout the whole length of the organ in a transversal manner, i.e., from the base to the lumen of the tubules. Transversal meiotic progression is also found in mammalian and in most insect testes, while in other meiotic models like *C. elegans,* meiosis progress longitudinally.

### Early recombination events precede synapsis in *D. magna*

To determine the timing and distribution of early recombination events by the appearance of Double Stranded Breaks (DSBs) in relation to synapsis, it was important to accurately establish the meiotic stages during prophase I. The antibody against SMC3 (a cohesin located underlying the AEs and LEs of the SC) is an ideal tool for this investigation. The SMC3 gene is present in the genome of *D. pulex* and it is transcriptionally active during meiosis (Colbourne et al. 2011; Schurko et al. 2009). Assuming that the role of SMC3 is conserved among *Daphnia* species, we concluded that it was a suitable marker to study *D. magna* synapsis.

SMC3 labelled the axial structures of *D. magna* chromosomes as early as leptotene and throughout all prophase I stages. We therefore established the timing of the appearance of proteins implicated in early recombination events depending on synapsis progression. We used γH2AX as a marker for early appearance of DSBs (Rogakou et al. 1998), and the recombinase Rad51, which is known to be recruited to DSBs downstream through the recombination pathway (Barlow et al. 1997). First, we began with a double immunolocalization of SMC3 and γH2AX. No γH2AX signals were detected in spermatogonia (data not shown). At early stages of prophase I (leptotene), γH2AX appeared as diffuse and intense accumulations distributed around the whole nucleus, while SMC3 labelled the AEs of the SC as light patched strands (Fig. 2a). At zygotene, short bright thick lines began to appear, presumably corresponding with the newly synapsed LEs (Fig. 2b). At pachytene, complete synapsis was observed with SMC3 labelling the LEs of the SC. Yet at this stage, the γH2AX signal was significantly reduced, appearing only like small foci that are located over SMC3 signals (Fig. 2c). At diplotene, SMC3 labelled the desynapsed LEs and the remaining LEs in retained synapsed areas, while γH2AX foci were barely observed (Fig. 2d) and were no longer detected at further stages. Overall, the SMC3 pattern of distribution during prophase I indicated that this cohesin component is located on the chromatin loops at early stages producing the observed diffuse nuclear labelling, and labels specifically the AE after leptotene, as previously described for mammals (Eijpe et al. 2003). At metaphase I, when all bivalents were perfectly aligned in the metaphase plate, SMC3 labelled the interchromatid domain, i.e., the region of contact between sister chromatids (Fig. 2e). Faint SMC3 signals were detected over chromosomes at metaphase II (Fig. 2f). Altogether, these results suggest that the early events of recombination precede the establishment of synapsis in *D. magna*.

Second, to better understand the relative timing of early recombination events in *D. magna* male meiosis, we performed a double immunolocalization of γH2AX and Rad51. As described above, γH2AX first appeared at the leptotene/zygotene transition as intense diffuse accumulations. At this stage, Rad51 labelling was undetected in some cells, which were presumably at leptotene and early zygotene stages (Fig. 3a), whereas in others, it appeared as small foci (Fig. 3b). These results suggest that, at early leptotene, no Rad51 was detected and recruited to DSBs labelled with γH2AX. Yet at late leptotene/zygotene, Rad51 began accumulating to the DBSs. Furthermore, when Rad51 foci intensified and became discrete at early pachytene, nuclei showed a light γH2AX staining (Fig. 3c), indicating that recombination events had progressed. After analysing 9 spermatocytes in pachytene, an average of 31 Rad51 foci were observed per cell, ranging from 27 to 33. This number is a preliminary estimate as serial confocal images are needed to verify this measurement in future studies. It is important to note that Rad51 foci were localized over or around γH2AX domains, but not entirely co-localized. In conclusion, γH2AX appears ahead of the appearance of Rad51 during prophase I. We can therefore frame early recombination events during the *D. magn*a meiotic prophase I stages. Unfortunately, crossover markers like the MLH1 antibody did not produce positive results. Therefore, we were unable to localize crossovers during *D. magna* spermatogenesis.

### Histone epigenetic modifications in *D. magna* meiosis

**Fig. 4:**
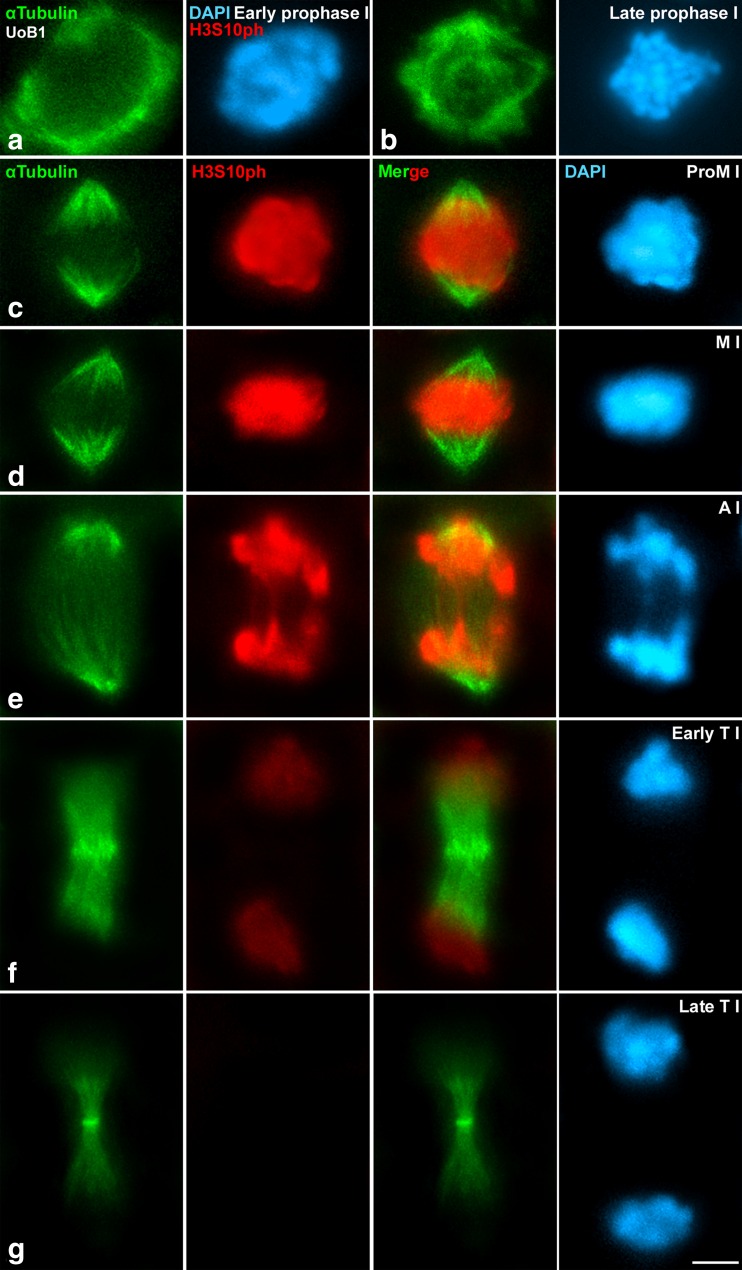
Epigenetic dynamics of H3S10ph. Double immunolabelling of αTubulin (*green*) to mark the spindle formation, and phosphorylation of serine 10 of histone 2, H3S10ph (*red*). Counterstaining of the chromatin with DAPI (*blue*). H3S10ph intensely labels the chromatin between prometaphase I and early telophase I, coinciding with stages of maximum level of condensation. **a** Early prophase I, **b** late prophase I. **c** Prometaphase I, **d** metaphase I. **e** Anaphase I, **f** early telophase I, **g** late telophase I. *Scale bar* in **f** corresponds to 2.5 μm

We immunolabelled two different variants of the histone H3 to analyse differences throughout meiosis. First, we labelled H3S10ph (commonly known as PH3), which is a widely used marker for mitotic and meiotic metaphase chromosomes in various animal species (Hendzel et al. 1997; Paula et al. 2013; Sakai et al. 2007; Sotero-Caio et al. 2011; Wei et al. 1998). The presence of this histone variant was previously reported by Western Blot in *D. magna* (Pijanowska and Kloc 2004), and by immunostaining in *D. pulex* females (Hiruta et al. 2010). By studying the immunostaining pattern of H3S10ph in *D. magna* male meiosis, we showed that serine 3 of H3 is also phosphorylated in this species. We therefore suggest that this histone post-translational modification is likely conserved among *Daphnia* species. To better indicate the progression of the cell cycle, we co-immunolocalized H3S10ph with αTubulin. We observed that histone 3 (H3) was not phosphorylated at serine 10 in premeiotic cells (data not shown) and during early (Fig. 4a) and late prophase I (Fig. 4b). As expected, no formed bipolar spindle was observed at prophase I. When bivalents were aligning during prometaphase I, the labelling of phosphorylated H3 intensified greatly (Fig. 4c) and similar labelling intensity was also detected when bipolar spindle was clearly appreciated at metaphase I (Fig. 4d). At early anaphase I, when homologous chromosomes began to segregate to opposite poles pulled by kinetochore microtubules detected with the anti-αTubulin antibody, the intense signal of H3S10ph was still observed (Fig. 4e). By early telophase I, where αTubulin was clearly detected at the interstitial microtubules, H3 began dephosphorylating (Fig. 4f). During late telophase I, phosphorylation of H3 disappeared and midbody detected by αTubulin helped mediating cytokinesis (Fig. 4g). Phosphorylation of H3 in serine 10 was also detected over the entire chromatin in metaphase II chromosomes, and disappeared at late telophase II. On the other hand, αTubulin staining, together with results with ACA labelling (see below), uncovered *D. magna* spindle dynamics. No previous data had been reported describing *D. magna* chromosomes as holocentric or monocentric. Our results show *D. magna* chromosomes as monocentric, since kinetochoric microtubules labelled by αTubulin reached only the centromeric areas (attaching to the kinetochores) in aligned bivalents and chromosomes in metaphase I and metaphase II, respectively.

**Fig. 5:**
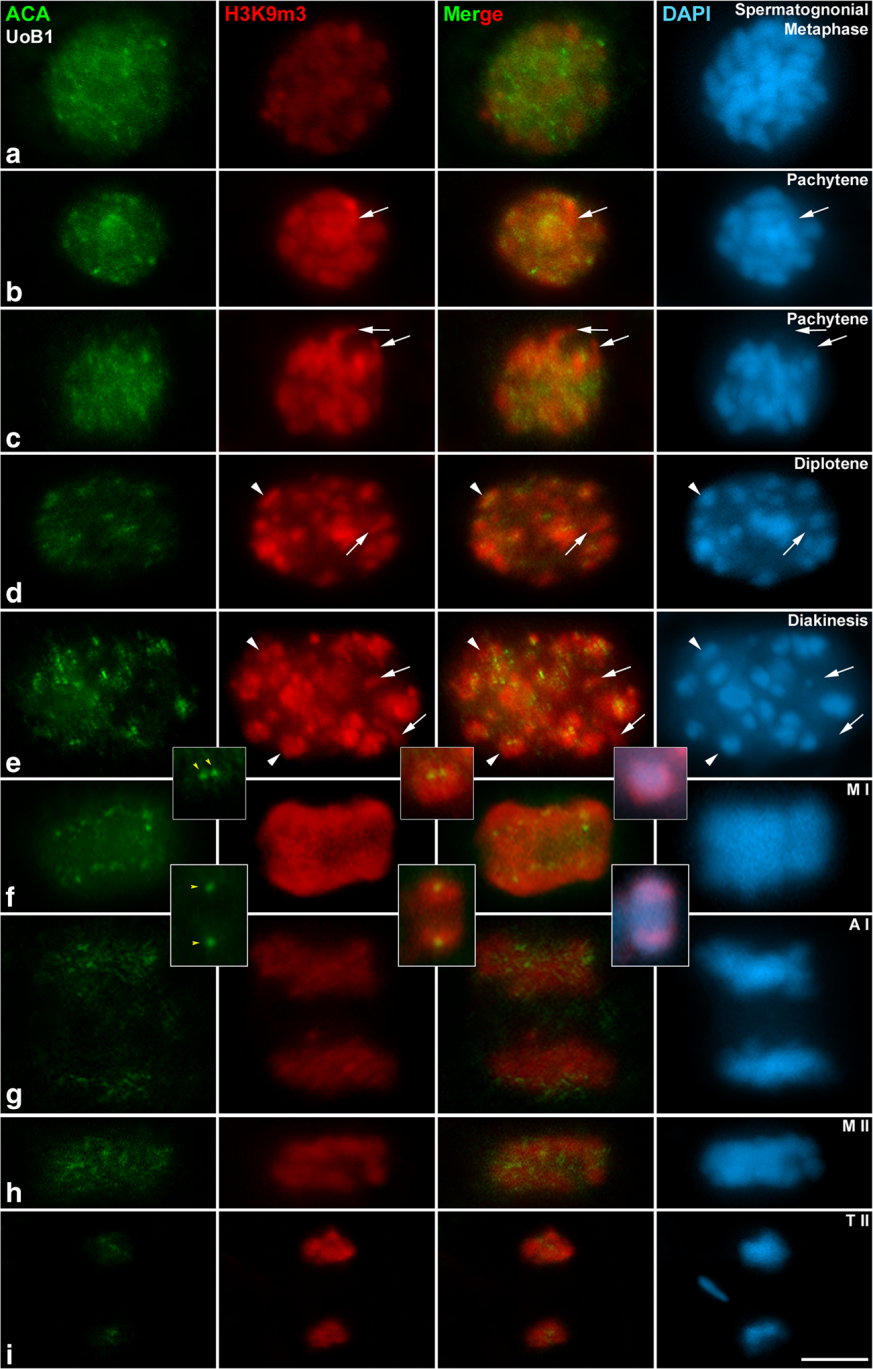
Epigenetic dynamics of H3K9m3. Double immunolabelling of ACA serum (*green*) as marker of kinetochores, and trimethylation of lysine 9 of histone 3, H3K9m3 (*red*). Counterstaining of the chromatin with DAPI (*blue*). H3K9m3 intensely labels the chromocentres during prophase I and a region that do not correspond to heterochromatin (*white arrows* in **b**–**d**). During metaphase I and metaphase II H3K93 labels the pericentromeric heterochromatin. The labelling pattern with ACA supports monocentric chromosomes in *D. magna.* The intensity of H3K9m3 signals decreases in telophase I and telophase II. **a** Spermatogonial metaphase, **b**–**c** pachytene, **d** diplotene, **e** diakinesis, a selected bivalent of the diakinesis is shown in the magnified inset. **f** Metaphase I, a selected bivalent of the metaphase I is shown in the magnified insets. **g** Anaphase I, **h** metaphase II, **i** telophase II. *White arrowheads* indicate some chromocentres in **d** and **f**. *Yellow arrowheads* indicate the kinetochores in *insets* of **e** and **f**. *Scale bar* in I corresponds to 2.5 μm

To detect the differences among epigenetic modifications of the same protein in *D. magna* male meiosis, we then immunodetected another post-translational modification of H3: the trimethylation of lysine 9 of the histone 3 (H3K9m3). Since this histone post-translational modification labelled heterocentromeric chromatin in other species, we co-immunolocalized with an anti-centromere antibody (ACA) that detects kinetochores. In premeiotic cells, at spermatogonial metaphase, we observed that H3K9m3 lightly labelled the centromeric heterochromatin, which correspond to the hyperchromatic DAPI areas in each chromosome (Fig. 5a). Already at prophase I, in pachytene, H3K9m3 brightly decorated the chromocentres (Fig. 5b), and one region that does not correspond to hyperchromatic DAPI signals (white arrows). The centromeric heterochromatin labelled with H3K9m3 partially co-localized with kinetochore signals stained with ACA. This same pattern was maintained at late pachytene (Fig. 5c). During diplotene, chromatin condensation increased, and chromocentres were easily detected with DAPI co-localizing with signals of H3K9m3 and partially overlapping with kinetochores (Fig. 5d). At diakinesis, condensation progressed and H3K9m3 was now intensely and clearly detected in chromocentres (Fig. 5e). One chromocentre is indicated with a white arrowhead in diplotene (Fig. 5d), and two chromocentres are similarly indicated in diakinesis (Fig. 5e). At diplotene (white arrows in Fig. 5d) and diakinesis (white arrows in Fig. 5e), there were also small areas of chromatin within the nucleus that were positively detected with H3K9m3, yet did not correspond to heterochromatin. At metaphase I, H3K9m3 labelled the centromeric heterochromatin of the bioriented bivalents (Fig. 5f). A selected bivalent at diakinesis and metaphase I is shown in the magnified insets of Fig. 5. The labelling pattern with ACA also supports the early conclusion of monocentric chromosomes in *D. magna*. The intensity of the H3K9m3 signal began to fade at telophase I, however, it was still detectable (Fig. 5g). At metaphase II, H3K9m3 again labelled the centromeric heterochromatin (Fig. 5h), and these signals persisted into telophase II (Fig. 5i). These results confirm that methylation of lysine 9 of histone 3 is consistently maintained throughout all stages of both meiotic divisions.

### Meiosis in inbred *D. magna* isolates

**Fig. 6:**
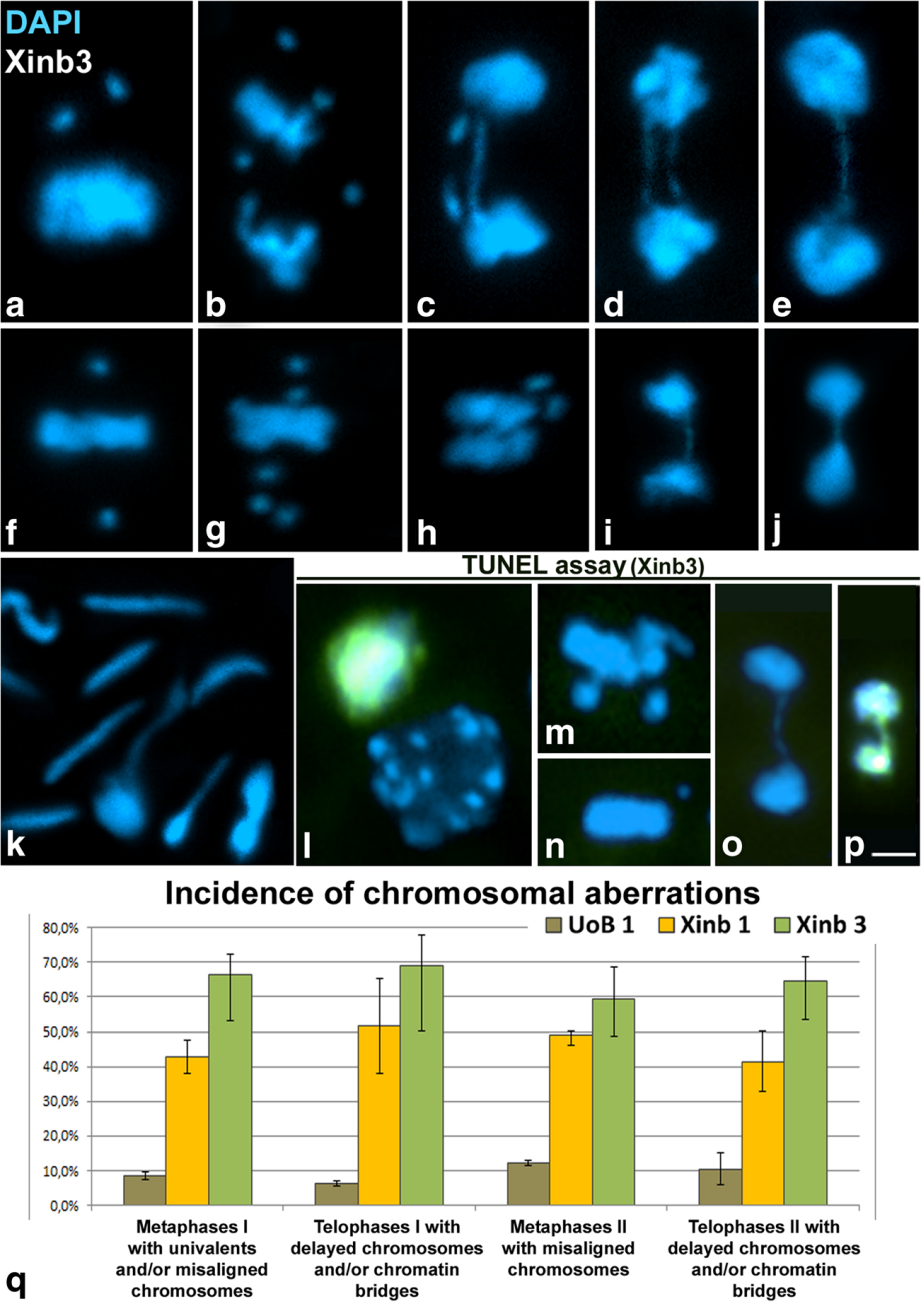
Male meiosis of inbred Xinb3 strain. DAPI-stained spermatocyte chromosomes from first **a**–**e** and second **f**–**j** meiotic divisions showing univalents/lagging chromosomes and/or chromatin bridges. Chromatin bridges were also observed during spermiogenesis at times (**k**). TUNEL assay of the same meiotic stages revealed some apoptotic cells in early and late prophase I (**l**–**p**). **a** metaphase I with univalents/misaligned chromosomes, **b** anaphase I with delayed chromosomes, **c**–**e** telophase I with chromatin bridges, **f**–**g** metaphase II with misaligned chromosomes, **h** early anaphase II with lagging chromosomes, **i**–**j** telophase II with chromatin bridges. TUNEL assay: **l** apoptotic zygotene and normal diakinesis. **m** Non apoptotic metaphase I with univalents, **n** non apoptotic metaphase II with misaligned chromosomes, **o** non apoptotic telophase I with chromatin bridges, **p** apoptotic telophase with chromatin bridges. *Scale bar* in P corresponds to 2.5 μm. **q** Graph representing the incidence of chromosomal aberrations. Metaphase I, telophase I, metaphase II and telophase II stages were observed in UoB1, Xinb1 and Xinb3 strains. Average percentages of chromosomal aberrations are shown in the graph. The number of individuals, the total number of cells observed (for each individual and in total), and the number of cells of each type of chromosomal aberration are shown in Table 1

After characterizing *D. magna* meiosis from the WT isolate (UoB1), we asked whether this meiotic phenotype could be generalized to other daphniid strains. We therefore examined the meiosis phenotype of males derived from the X strain, which is the strain used to produce the Xinb3 isolate for the ongoing *D. magna* genome project, thrice inbred and characterized to be relatively infertile (Routtu et al. 2010). We aimed to describe the cytological characterization of that relative infertility compared to less inbred strains. After staining with DAPI squashed testes from Xinb3 natural males, we observed clear chromosomal aberrations in both meiotic divisions, and morphological aberrations during spermiogenesis. Xinb3 spermatocytes showed no apparent aberrations during prophase I stages, as pairing and synapsis followed by inmunodetecting SMC3 showed an identical distribution of the one previously shown for UoB1. However, our observations in later stages indicated clear defects in these inbred lines, suggesting that recombination is somehow also defective. Although chromosome condensation was apparently not affected, we found different defects in chromosome behaviour. We observed metaphases I with misaligned bivalents or univalents (Fig. 6a). Although univalents often move to the poles before or after the rest of the chromosomes, the small chromosome sizes in *D. magna* did not allow such a distinction to be made. Xinb3 anaphase I/telophase I spermatocytes showed one to four lagging chromosomes and/or chromatin bridges (Fig. 6b–e). Misaligned chromosomes were often observed outside the metaphase II plate (Fig. 6f–g), and anaphase II/telophase II also clearly showed lagging chromosomes and chromatin bridges (Fig. 6h–j). These results suggest that inbreeding of the X strain to produce Xinb3 promotes defects in chromosome segregation. Moreover, inbreeding also affects spermiogenesis, since some aberrant mature spermatids were detected (Fig. 6k).

After analysing our results, we asked if Xinb3 chromosomal aberrations may induce cell arrest, and if these degenerating cells could also undergo apoptosis. We consequentially carried out a TUNEL assay on squashed testes of Xinb3 natural males, which detects DNA fragmentation associated with programmed cell death. We detected only basal levels of apoptosis (average 1–2 % of the total cells), and no apparent apoptosis was detected in cells showing chromosomal aberrations. No apoptosis was detected throughout prophase I (see, for example, diakinesis nucleus in Fig. 6l). Moreover, no TUNEL labelling was detected during the vast majority of the metaphases I with univalents, metaphases II with misaligned chromosomes, and telophases from the first and second meiotic division (Fig. 6m–o). However, we sporadically observed spermatocytes with bright, small and hypercondensed nuclei undergoing apoptosis, as marked with TUNEL dUTP-fluorescein nick end labelling (zygotene in Fig. 6l and telophase I in Fig. 6p), in line with previous findings showing that dividing spermatocytes showing hypercondensed chromosomes are apoptotic (Adelman and Petrini 2008). The small fraction of cells undergoing apoptosis in Xinb3 could be attributed to basal rate common in any proliferative tissue, and was equal to the rate observed in the wild-type populations. TUNEL assays were also performed using UoB1 samples, which revealed no significant differences in comparison with Xinb3 (Supplementary Fig. 3). These results demonstrate that although the Xinb3 isolate shows high incidence of chromosome dynamic aberrations, meiotic progression was not arrested.

To better understand the incidence rate of these chromosomal aberrations, we quantified the appearance of the detected aberrations comparing UoB1 males with males of Xinb1 and Xinb3 isolates (Fig. 6q and Table 1). We analysed both testes from five individuals per line. We made two preparations per testis to maximize the spreading of the material and to obtain quality images of the cells. For greater accuracy, we selected preparations from individuals that possessed a high number of dividing spermatocytes. For each individual, we counted between 15 to 41 cells per stage (a general average of 25–30 cells per stage and per individual). The proportion of cells containing chromosomal aberrations in metaphase I, telophase I, metaphase II and telophase II for the UoB1, Xinb1 and Xinb3 isolates are shown in Table 1, along with the total number of cells counted by line and the average per individual. More detailed information is presented in the supplementary material (Supplementary Tables 1, 2 and 3 for UoB1, Xinb1 and Xinb3, respectively). In conclusion, we observed increasing numbers of metaphase I with univalents, telophases I with bridges, metaphases II with misaligned chromosomes and telophases II with comparing the inbred isolates (Xinb1 and Xinb3 respectively) with the natural population (UoB1).

**Table 1:**
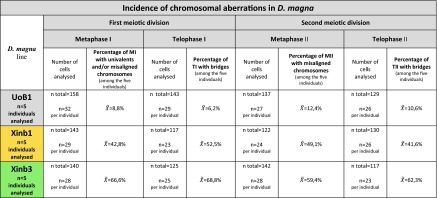
Incidence of chromosomal aberrations in *Daphnia magna* lines UoB1, Xinb1 and Xinb3

*n total* total number of cells analysed per stage in the correspondent *D. magna* line

$\overset{\sim }{X}$ average of the percentages of incidence of the correspondent chromosomal aberrations per stage and per *D. magna* line

### Sperm quality analysis

**Fig. 7:**
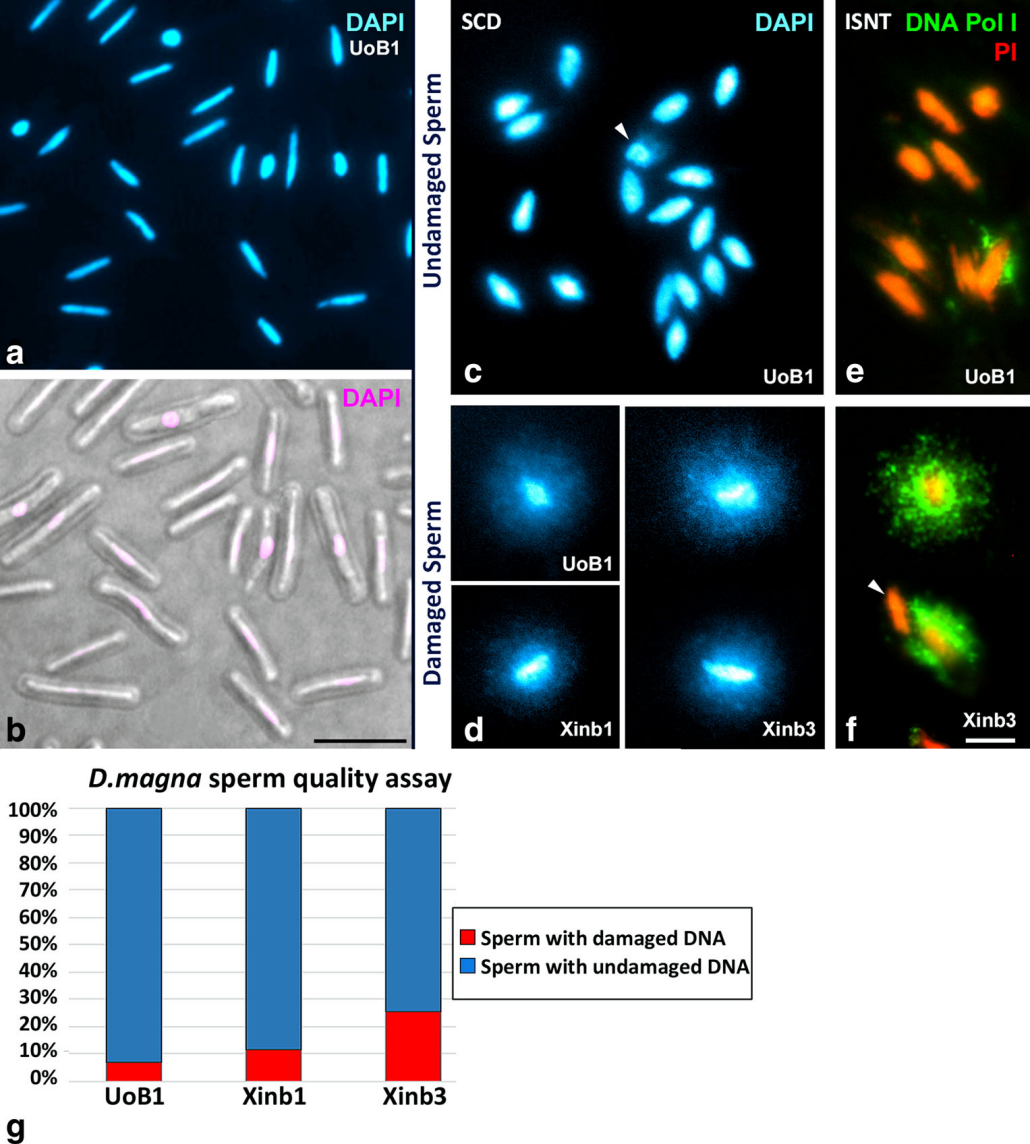
Detection of DNA damage in Xinb3 sperm. *Daphnia magna* sperm (**a**–**b**). **a** UoB1 spermatozoa with DNA counterstained with DAPI **b** UoB1 spermatozoa shown with DIC microscopy and pseudo-coloured DAPI in pink. Sperm Chromatin Dispersion (SCD) technique (**c**–**d**). **c** UoB1 sperm with undamaged DNA in UoB1. **d** Multiple undamaged sperm can be seen while there is one spermatozoid that possesses slight damage (white arrowhead). Examples of sperm with damaged DNA in UoB1, Xinb1 and Xinb3 in **d**. In situ Nick translation (ISNT) technique (**e**–**f**). **e** UoB1 sperm with undamaged DNA. **f** Xinb3 sperm with damaged DNA. Multiple undamaged sperm can be seen while there is one spermatozoid that possesses slight damage in **e** (*white arrowhead*). One undamaged spermatozoid can be observed next to the damaged ones in **f** (*white arrowhead*). *Scale bar* in **f** corresponds to 2.5 μm. **g** Graph representing the percentages of damaged versus undamaged spermatozoa in UoB1, Xinb1 and Xinb3. The number of individuals, the number spermatozoa analysed, and the percentages of damaged versus undamaged spermatozoa are shown in Table 2

After observing severe failures in chromosomal segregations in both meiotic divisions in the inbred Xinb3 isolate, and after confirming that the majority of the cells carrying these aberrations did not enter apoptosis, we then asked if the spermatozoa of Xinb3 carried DNA damage, since sperm production in these males is not completely arrested. We therefore carried out a Sperm Chromatin Dispersion test, using the HaloTech® kit that was previously used for other species (Enciso et al. 2006; Fernandez et al. 2003). Spermatozoa from the UoB1, Xinb1 and Xinb3 natural males were first examined by contrast phase light microscopy and counterstained with DAPI to analyse their natural appearance. *D. magna* spermatozoa are composed of a very compact elongated nucleus surrounded by a protective capsule of undetermined material, lacking flagella or pseudopodia-like extensions and with a length of around 9–11 μm (Fig. 7a–b), similarly to those previously described for *D. carinata* males (Zhang et al. 2005). Five animals of each line were analyzed. As thousands of spermatozoa are found from a single ejaculate, an accurate estimate is easily obtained. We counted around 300 randomly picked spermatozoa per sample. Each were scored as undamaged spermatozoid or spermatozoid with damaged DNA (Fig. 7g). In total, an average of around 1500 spermatozoa were analysed per line. When undamaged, spermatozoids were characterized by a round central core with eventually very small and compact surrounding haloes (Fig. 7c). By contrast when spermatozoa had DNA damage, nucleoids with a central core and a large peripheral halo of dispersed DNA fragments were observed (Fig. 7d). To corroborate our results, the presence of DNA breaks in the dispersed haloes was validated by means of direct incorporation of labelled nucleotides with in situ Nick Translation technique (Fig. 7e–f). Upon quantifying the results for each sample, we concluded that males of the UoB1 isolate, which we consider a natural outbred isolate, showed a very low fraction of damaged spermatozoa, 6.7 %. By contrast, naturally obtained males from the inbred Xinb1 and Xinb3 isolates showed a significant higher fraction of spermatozoa with DNA damage, 11.4 and 25.6 % respectively (Fig. 7g). The number of spermatozoa counted per individual, and the average percentages of spermatozoa with undamaged DNA are shown in Table 2. The complete detailed information of the sperm quality analysis is shown in the supplementary material (Supplementary Table 4).

**Table 2:**
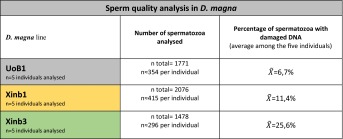
Sperm quality analysis in *Daphnia magna* lines UoB1, Xinb1 and Xinb3

*n total* total number of spermatozoa analysed in the correspondent *D. magna* line

$\overset{\sim }{X}$ average of the percentages of spermatozoa with damaged DNA in the correspondent *D. magna* line

## Discussion

### The timing of meiosis in *D. magna*

This study (1) demonstrates that *D. magna* male meiosis progresses transversally in the testis, (2) cytologically identifies the meiotic stages and (3) determines the progression for some of the most important events in meiosis: synapsis and early recombination. Prior to this study, comparative data for Crustacea regarding the interdependence of recombination and synapsis were lacking. One of two general models is likely for *Daphnia*. Recombination may precede the full formation of the SC such as in budding yeast (Kleckner 1996), *Arabidopsis* (Grelon et al. 2001), mouse (Mahadevaiah et al. 2001) and grasshoppers (Viera et al. 2004). In these species, the earliest defined molecular event in meiotic recombination occurs with the formation of DSBs (in leptotene). Alternatively, the formation of the SC may precede recombination as in *Drosophila* (McKim et al. 1998) and *C. elegans* (Dernburg et al. 1998). Either the *grasshopper model* or the *fly model* would have been expected for *Daphnia* species. Our study demonstrates that the initiation of recombination in *D. magna* occurs before the initiation of synapsis. This was detected by the formation of DSBs (labelled by γH2AX), which diminished with synapsis progression. We also observed that Rad51 foci appeared downstream of DSB formation, immediately after γH2AX. Interestingly, no specific differential signal labelled by γH2AX was visible in any particular chromosome or chromosome pair during prophase I in *D. magna*, as is the case for the X chromosome in grasshoppers (Viera et al. 2004), and the XY bivalent in mice (Fernandez-Capetillo et al. 2003; Mahadevaiah et al. 2001) and in eutherian mammals (de la Fuente et al. 2007). This pattern can be explained by *Daphnia* lacking sex chromosomes; sex determination is instead environmentally determined (Kleiven et al. 1992). Thus, the progression of recombination (followed with γH2AX and Rad51) is temporally and spatially correlated with synapsis (labelled by SMC3). Hence, our results indicate that meiosis in male *D. magna* is similar to the *grasshopper model.* This new finding within a phylogenetic context suggests that the alternative mechanism shared by *D. melanogaster* and *C. elegans* evolved independently in both lineages. However, other important players implicated in meiotic crossovers (proteins such as MLH1/3, HEI10/RNF212 or CDK2) (Zickler and Kleckner 2015) should also be studied to complete our understanding of *Daphnia* meiotic recombination.

Molecular studies are needed to specify the recombination pathways that trigger the formation of a presumed SC in *D. magna* to allow synapsis. SC components have not yet been fully characterized in *Daphnia* species, since homologous SC protein sequences rapidly diverge among species. However, a potential homologue of SYCE2—a small protein specifically located at the central element (CE) of the SC (Hamer et al. 2006)—was identified in the *D. pulex* genome, despite its very long branch along the phylogenetic tree leading to *Daphnia* (Fraune et al. 2012). Although this gene is present and transcribed in the *Daphnia* genome, additional evidence is required to ensure the presence of SC in *Daphnia*. Nonetheless, we speculate that SC components and their dynamics may play an important role during spermatogenesis in *Daphnia* males, possibly also in the transition from parthenogenesis to sexual oogenesis in females, since SC proteins are meiotic-specific and must be tightly regulated to allow synapsis during meiotic progression. In this regard, it was suggested that *D. pulex* parthenogenesis is an abortive female meiosis in anaphase I, in which meiosis progresses normally during prophase I, and that bivalents may be observed in the metaphase I plate (Hiruta et al. 2010). If so, future studies will need to determine the similarities or differences between the regulation of the SC in female and male *Daphnia* meiosis.

### *D. magna* males as a model for epigenomics

*Daphnia* offers many benefits for the study of epigenetics (Harris et al. 2012). Histone epigenetic modifications are also good cytological markers with which to estimate the regulation of specific chromosome domains within a certain time in the cell cycle. Hence, our second approach to characterize meiosis in *D. magna* consisted of detecting specific histone post-translational modifications in the testis. Previously, we described the role of phosphorylation of Ser139 modification of H2A in the structural histone variant H2AX (γH2AX) in *D. magna* male meiosis. We suggested that γH2AX is an excellent marker for early recombination events in *Daphnia* by labelling DSBs—a method that worked well when studying mammals (Mahadevaiah et al. 2001) and grasshoppers (Viera et al. 2004). On the other hand, previous studies revealed the implication of γH2AX in meiotic sex chromosome inactivation (Fernandez-Capetillo et al. 2003), and in the meiotic silencing of unsynapsed chromatin (Turner et al. 2004). As *Daphnia* have no sex chromosomes per se, we cannot presume a similar role for this histone post-translational modification. Besides, studies in grasshopper species have shown that γH2AX also appears at the centromeres during meiotic metaphase I and anaphase I, somehow regulating centromere behaviour (Cabrero et al. 2007). As we did not detected γH2AX at the centromeres during our study, we can only point out a role of γH2AX during the early steps of meiotic recombination.

We then studied if different post-translational modifications of H3 had different patterns of localization. H3 is phosphorylated in Serine 10 by Aurora B kinase and regulates the dynamic condensation/relaxation of chromosomes, chromatid cohesion, as well as gene expression (Cerutti and Casas-Mollano 2009). During meiosis, H3S10ph is phosphorylated in all organisms studied to date (Feitoza and Guerra 2011; Kaszas and Cande 2000; Paula et al. 2013; Speliotes et al. 2000; Staiber 2012; Wei et al. 1998). We observed that in *D. magna*, H3S10ph labels the entire chromatin between prometaphase and early telophase in both meiotic divisions. This pattern differs from the one observed in all other arthropods. For instance in *D. melanogaster*, H3S10ph in spermatocytes displays a spotted distribution all over the chromatin at every meiotic stage (Hennig and Weyrich 2013). *D. magna* H3S10ph pattern also differs from those of other insects, where the onset of this histone post-translational modification initiates at diplotene, showing a similar timing for grasshoppers (Orthoptera), bugs (Hemiptera), and beetles (Coleoptera) (Sotero-Caio et al. 2011). The fact that all these species possess sex chromosomes—and that from middle diplotene on, those sex chromosomes are always hypophosphorylated in relation to the autosomes in insects (Sotero-Caio et al. 2011) including *D. melanogaster* (Hennig and Weyrich 2013)—suggests that in these species, this epigenetic modification must be previously promoted to facilitate a differential regulation of the pair of sex chromosomes in meiosis. By contrast in *Daphnia*, where no true sex chromosomes exist, the phosphorylation of H3 can be triggered in all chromosomes at the same time, concomitantly with chromosome congression in prometaphase I/II, and persist at the highest levels of condensation in metaphase I/II, presumably playing a role in regulating chromatin condensation. H3S10ph is therefore an ideal marker of metaphase in *Daphnia*, and thus of high level of chromatid condensation, pointing at this histone post-translational modification as an evolutionary conserved epigenetic marker for cells under division. This hypothesis offers an excellent tool to be applied for phenotypic plasticity studies in *Daphnia* species. In other words, cell proliferation in inducible defences such as helmets and neckteeth (Laforsch and Tollrian 2004) and cell division related to embryogenesis and development and tissue regeneration could be cytologically detected and followed by labelling this particular histone variant.

Finally, we analysed trimethylation of histone 3 in lysine 9 (H3K9me3), originally studied because of its binding by Heterochromatin Protein 1a (HP1a), which is an essential step in the establishment of heterochromatin (Bannister and Kouzarides 2011). When analysing its distribution pattern in *Daphnia*, we detected H3K9m3 at chromocentres during prophase I and at the heterochomatic chromatin in metaphase I and II. This data are in agreement with previous observations obtained from organisms ranging from yeasts to mammals, demonstrating that the methylation state is closely linked to transcriptional activity. The fact that H3K9m3 has been found at heterochromatic regions in the freshwater crustacean *Asellus aquaticus* (Barzotti et al. 2006) suggests that this histone post-translational modification may have a similar evolutionary conserved mechanism of regulating DNA among crustaceans. H3K9m3 localization to heterochromatic defines constitutive heterochromatin that contains permanently silenced genes in genomic regions such as the centromeres and telomeres (Trojer and Reinberg 2007). Moreover, H3K9m3 was implicated in epigenetic silencing and constitutive heterochromatin (Schotta et al. 2004). In addition, H3K9m3 participates in the meiotic sex chromosome inactivation in the germ line (Khalil and Driscoll 2006). The fact that we also find labelling of H3K9m3 in regions of the chromatin that do not correspond to hyperchromatic DAPI areas, suggests that further studies are needed in *D. magna* to determine if H3K9 methylation could be playing a role in silencing specific chromosomes or chromosome regions outside heterochromatin domains, which might be regulating sex determination. However, we cannot exclude the possibility that we are observing heterochromatic regions that are not rich in AT, as it is known that DAPI preferentially binds the minor groove of DNA in AT-rich regions. In addition, our results with H3K9me3 and kinetochoric signals (ACA) also support the new finding that *D. magna* possess monocentric chromosomes. These results offer an excellent tool to locate centromeric heterochromatin, which could be very useful in studying chromosomal reorganizations such as massive deletions, duplications or translocations, which have been previously reported in *Daphnia* species (Tucker et al. 2013; Xu et al. 2011).

It is important to note that histone modifications do not occur in isolation, but rather in a combinatorial manner (Nowak and Corces 2004). As a related example, H3S10ph may have a role at regulating H3K9 methylation in human cells (Duan et al. 2008), illustrating the complexity of histone modifications. Further studies will need to be conducted to explain which histone post-translational modifications are responsible for chromosome condensation or gene silencing, and interactions (synergies or steric competition) among different variants could also be evaluated for possible functional interrelations during cell cycle progression.

### *Daphnia* male fertility

Having characterized *D. magna* male meiosis, we wished to understand the potential causes for defective sperm production, which may be a result of inbreeding. In the case of *Daphnia*, inbreeding can be rapid, by mating genetically identical males and females of the same isolate, thereby exposing recessive or deleterious alleles in homozygous state. It is well known that inbred animals are less likely to survive and less likely to reproduce than animals of outbred populations (Charlesworth and Willis 2009). Inbreeding depression results in the loss of biological fitness and vigour, smaller litter/clutch sizes and decreased body size, developmental disruption, lower birth rate, higher juvenile mortality, shorter life span, and increased expression of inherited disorders. Studies from social insects, mites, and spiders revealed the profound impacts of inbreeding on the fitness, the physiology, and the behaviour of arthropods as well (Tabadkani et al. 2012). Inbreeding depression is also a well-known phenomenon in *Daphnia* species (Deng and Lynch 1997; Haag et al. 2002).

The *Daphnia* Xinb inbred line is reported to be partially infertile (Routtu et al. 2010) and our investigations revealed that this infertility may be a consequence of inbreeding using sperm quality as an indicator of fertility. When analysing recursively inbred isolates Xinb1 (one round) and Xinb3 (three rounds) compared to a wild-type population (UoB1), we discovered that inbreeding leads to increasingly severe fertility problems that are possibly related to chromosomal aberrations, by showing an association between unresolved segregation mistakes in anaphase I and II with the level of induced homozygosity of the genome by inbreeding. These segregation aberrations may also be due to previous errors during prophase I, i.e. errors in recombination, as our analysis also indirectly shows that the *meiotic pachytene checkpoint* (Zickler and Kleckner 2015) may not be very efficient in *D. magna.* We suggest that the damaged spermatozoa in the inbred lines could carry aneuploidies lacking or exceeding one or more chromosomes due to the previous recombination or segregating defects. Given that the chromosomal aberrations bypassed meiotic checkpoints and did not induce programmed cell death in the Xinb3 trials, we propose that secondary spermatocytes and spermatids may be accumulating DNA damage and aneuploidies, which could explain this isolate’s reported infertility (Routtu et al. 2010).

Surprisingly, natural *Daphnia* populations (UoB1) also show chromosomal aberrations (albeit minimal compared to the inbred strain). An explanation may be that chromosome aberrations (such as paracentric inversions) accumulate over parthenogenesis with little to no consequences, but negatively impacts the animals’ fitness once sexual reproduction is resumed.

We hereby suggest a new method to measure *Daphnia* male fertility, by having showed that the chromosomal aberrations associated with inbreeding produces sperm containing DNA damage. As the protocol presented here detects DNA damage but does not decipher the origin of the observed segregation errors, it could be combined with other studies (such as the analysis of the crossover status in both wild-type and inbred lines) to better understand fertility problems. It is worth emphasizing that this methodology is a novel and easy protocol to measure *Daphnia* sperm quality, and can therefore be used to explore *Daphnia* male fertility among divergent lineages, obligate asexual lines, or among populations subjected to environmental toxic compounds (pollution) in relation to chemical risk assessment and ecosystem health.

Sperm structure and morphology have a major impact on the success rate of fertilization; several studies have indicated the importance of DNA and chromatin status related with fertilization success (Agarwal and Said 2003). In this sense, our results suggest that in *Daphnia*, possible recombination and segregation failures during meiosis of inbred lines can lead to aneuploidy and persistence of DNA damages in the male gametes. This and future work, adds meiotic cytology to the research toolkit for model species *Daphnia*, which also serves as a model arthropod crustacean for comparative cytobiology, especially given the animal’s unique attributes.

## Electronic supplementary material

Below is the link to the electronic supplementary material.

Supplementary Fig. 1 Immunoblot analysis of *Daphnia magna* testis protein extracts. SMC3, αTubulin, H3S10ph, H3K9m3, γH2AX and Rad51 are detected in bands of the expected molecular weight. The positions of molecular mass markers (Mr K) are indicated. The expected relative migration distances (MW) are as follows: SMC3 ~ 140 kDa, αTubulin ~ 50 kDa, H3S10ph ~ 15 kDa, H3K9m3 ~ 17 kDa (Pijanowska and Kloc 2004), γH2AX ~ 15 and Rad51 ~ 37 kDa. (GIF 76 kb)

High resolution image (TIF 861 kb)

Supplementary Fig. 2 Reconstruction of *Daphnia magna* testis. **a** Counterstaining with DAPI. **b** DIC image for the whole organ. These images are a z projection of several focal planes through the organ volume. The histology of *D. magna* testis mainly consists on a tubule containing polyploid cells and spermatogenic cells immersed in connective tissue. Meiosis progress throughout the whole length of the organ in a transversal manner. Polyploid cells and spermatogonia are located at the base of the tubule (a, b). Primary spermatocytes (c, d) undergo first meiotic division, and they develop into secondary spermatocytes (e). Their division results in the formation of the spermatids (f), which lie in the luminal part of the tubule. Mature spermatozoa accumulate at the lumen of the testis. A top, middle and bottom planes are shown for the middle zone of the testis. The serial sections demonstrate that polyploid cells and primary spermatocytes are located at the basis of the testis since they are observed only at the top and bottom focal planes, whether spermatozoa are accumulated in the lumen along the entire length of the testis and thereby mostly observed in the middle planes. A z projection of the posterior region of the testis is also shown. *Scale bar* corresponds to 0.2 mm. (GIF 161 kb)

High resolution image (TIF 1930 kb)

Supplementary Fig. 3 TUNEL assay in UoB1. Spread of UoB1 Spermatocytes. DNA counterstained with DAPI (blue) and TdT-mediated dUTP-fluorescein nick end-labelling detecting DNA fragmentation-associated apoptosis of spermatocytes (green). The field shows several spermatocytes in prophase I (some of them indicated by a yellow star), and also dividing spermatocytes (some of them indicated by a red star). Only one spermatocyte in prophase I is detected as apoptotic in this field (white arrow). Scale bar corresponds to 10 μm. (GIF 388 kb)

High resolution image (TIF 11891 kb)

Supplementary Table 1 Detailed information of the study of the incidence of chromosomal aberrations in *Daphnia magna* line UoB1*. (DOCX 18 kb)*

Supplementary Table 2 Detailed information of the study of the incidence of chromosomal aberrations in *Daphnia magna* line Xinb1. (DOCX 18 kb)

Supplementary Table 3 Detailed information of the study of the incidence of chromosomal aberrations in *Daphnia magna* line Xinb3*. (DOCX 20 kb)*

Supplementary Table 4 Detailed information of the sperm quality analysis in *Daphnia magna* lines UoB1, Xinb1 and Xinb3*. (DOCX 18 kb)*

Supplementary Video 1 Metaphase I labelled with αTubulin. Immunolabelling of αTubulin (green) and counterstaining of the chromatin with DAPI (blue). All bivalents are perfectly aligned in the metaphase plate and a bipolar spindle is formed. (MOV 1825 kb)

Supplementary Video 2 Telophase I labelled with αTubulin. Immunolabelling of αTubulin (green) and counterstaining of the chromatin with DAPI (blue). Homologous chromosomes have migrated to opposite poles and the contractile ring of cytokinesis between both daughter cells is being formed. (MOV 1471 kb)

Supplementary legend of Fig. 1 *Spermatogonias* are the primordial cells, which divide through mitosis to maintain a constant backup of cells. Their nuclei are big and possess numerous chromocentres that are clearly observed as hyperchromatic areas with DAPI (Fig. 1c). As chromatin condensation takes place, chromosomes begin to congress into the metaphase plate in *spermatogonial prometaphase* (Fig. 1d). When chromosomes are fully condensed and aligned, we observe *spermatogonial metaphases* in polar (Fig. 1e) or lateral (Fig. 1f) views. These spermatogonial metaphases are undergoing mitotic division, and therefore have 20 aligned chromosomes in *D. magna*. Identical sister chromatids of each chromosome are segregated during *spermatogonial anaphase* (Fig. 1g). After completing mitosis, some of the spermatogonias enter meiosis. During the first meiotic division, the *prophase I* stage lasts the longest. We identify *leptotene/zygotene* stages as small nuclei with homogeneous and hypochromatic chromatin (Fig. 1h). In *pachytene*, the chromatin has already begun condensation and the chromocentres—corresponding to the heterocentromeric chromatin surrounding the centromeres—are visible (Fig. 1i). In *diplotene*, the nuclear size is increased and chromocentres are clearly distinguished (Fig. 1j). In *diakinesis*, nuclei significantly increase in volume (Fig. 1k). Chromatid condensation and bivalent alignment take place during *prometaphase I* (Fig. 1l). In *metaphase I*, ten bivalents are perfectly aligned in the metaphase plate although the highly condensed chromatin does not allow the identification of each single one (Fig. 1m). To corroborate the alignment of the bivalents, we immunodetected these cells with αTubulin and we clearly observe a bipolar spindle (supplementary video 1). Homologues segregate during early *anaphase I* (Fig. 1n) and begin migrating to the cell poles in late anaphase I (Fig. 1o). Chromatin decondensation begins in early *telophase I* when homologues reach opposite poles (Fig. 1p). In late telophase I, chromosome segregation is complete and we assume that the nuclear envelope begins to reconstitute when we observe round nuclei in opposite poles (Fig. 1q). Using αTubulin, we observe the contractile ring of cytokinesis between both daughter cells, demonstrating that these cells are indeed at the telophase stage (supplementary video 2). Each of these cells will progress to *interkinesis* (Fig. 1r), an intermediate stage between both meiotic divisions when no DNA replication occurs. During *prophase II,* chromatin begins to condense and the nuclear envelope is disintegrated at the stage’s conclusion. Ten chromosomes congregate during *prometaphase II* (Fig. 1s) and finally align into the metaphase II plate (Fig. 1t). Sister chromatids will segregate to opposite poles during anaphase II (Fig. 1u) and the nuclear envelope will be reconstituted concomitantly with chromatin decondensation in *telophase II* (Fig. 1v). Each of the haploids cells obtained after completion of meiosis, called early spermatids (Fig. 1w), would enter a maturation process called *spermiogenesis*, where chromatin will be highly compacted and nuclei shape will change from round to elongate in the medium spermatids (Fig. 1x). Nuclei will again decrease in size to form mature spermatozoa (Fig. 1y). (DOCX 13 kb)

## Abbreviations

ACA: Anti-centromere antibody
AE: Axial element of the synaptonemal complex
DSB: Double strand break
γH2AX: Phosphorylated histone H2AX
H3S10ph: Histone 3 phosphorylated in the Serine 10
H3K9m3: Histone 3 trimethylated in the Lysine 9
LE: Lateral element of the synaptonemal complex
SC: Synaptonemal complex
SCC: Sister chromatid cohesion
SMC3: Structural maintenance of chromosomes
TUNEL: TdT-mediated dUTP-fluorescein nick end labelling
