# Supplementary figures and images for: Male meiosis in Crustacea: synapsis, recombination, epigenetics and fertility in Daphnia magna
Source: Chromosoma. 2015 Dec 21;125(4):769–87. doi: 10.1007/s00412-015-0558-1 (PMC5023733; doi:10.1007/s00412-015-0558-1)

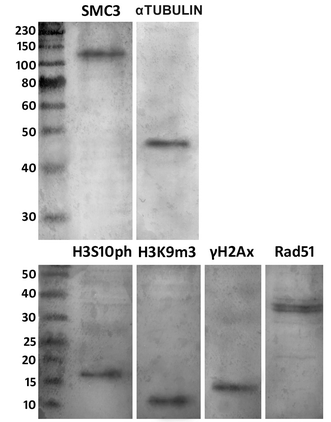

Supplement: Supplementary file 1 — Immunoblot analysis of Daphnia magna testis protein extracts. SMC3, αTubulin, H3S10ph, H3K9m3, γH2AX and Rad51 are detected in bands of the expected molecular weight. The positions of molecular mass markers (Mr K) are indicated. The expected relative migration distances (MW) are as follows: SMC3 ~ 140 kDa, αTubulin ~ 50 kDa, H3S10ph ~ 15 kDa, H3K9m3 ~ 17 kDa (Pijanowska and Kloc 2004), γH2AX ~ 15 and Rad51 ~ 37 kDa. (GIF 76 kb) [file 412_2015_558_Fig10_ESM.gif]

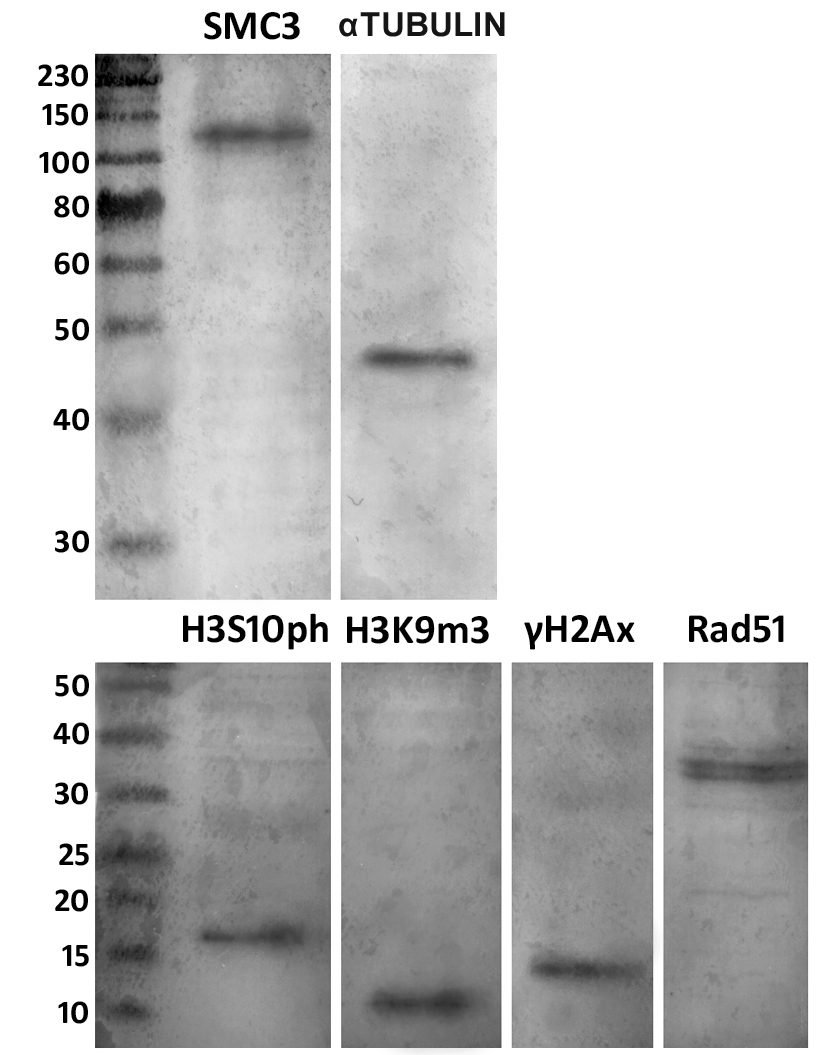

Supplement: Supplementary file 2 — High resolution image (TIF 861 kb) [file 412_2015_558_MOESM1_ESM.tif]

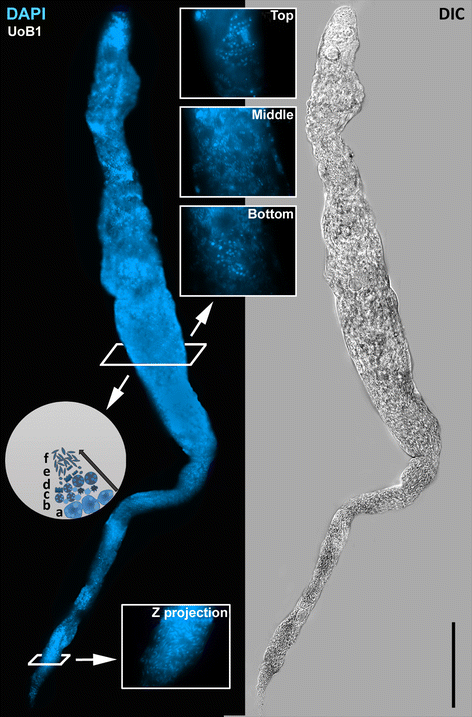

Supplement: Supplementary file 3 — Reconstruction of Daphnia magna testis. a Counterstaining with DAPI. b DIC image for the whole organ. These images are a z projection of several focal planes through the organ volume. The histology of D. magna testis mainly consists on a tubule containing polyploid cells and spermatogenic cells immersed in connective tissue. Meiosis progress throughout the whole length of the organ in a transversal manner. Polyploid cells and spermatogonia are located at the base of the tubule (a, b). Primary spermatocytes (c, d) undergo first meiotic division, and they develop into secondary spermatocytes (e). Their division results in the formation of the spermatids (f), which lie in the luminal part of the tubule. Mature spermatozoa accumulate at the lumen of the testis. A top, middle and bottom planes are shown for the middle zone of the testis. The serial sections demonstrate that polyploid cells and primary spermatocytes are located at the basis of the testis since they are observed only at the top and bottom focal planes, whether spermatozoa are accumulated in the lumen along the entire length of the testis and thereby mostly observed in the middle planes. A z projection of the posterior region of the testis is also shown. Scale bar corresponds to 0.2 mm. (GIF 161 kb) [file 412_2015_558_Fig11_ESM.gif]

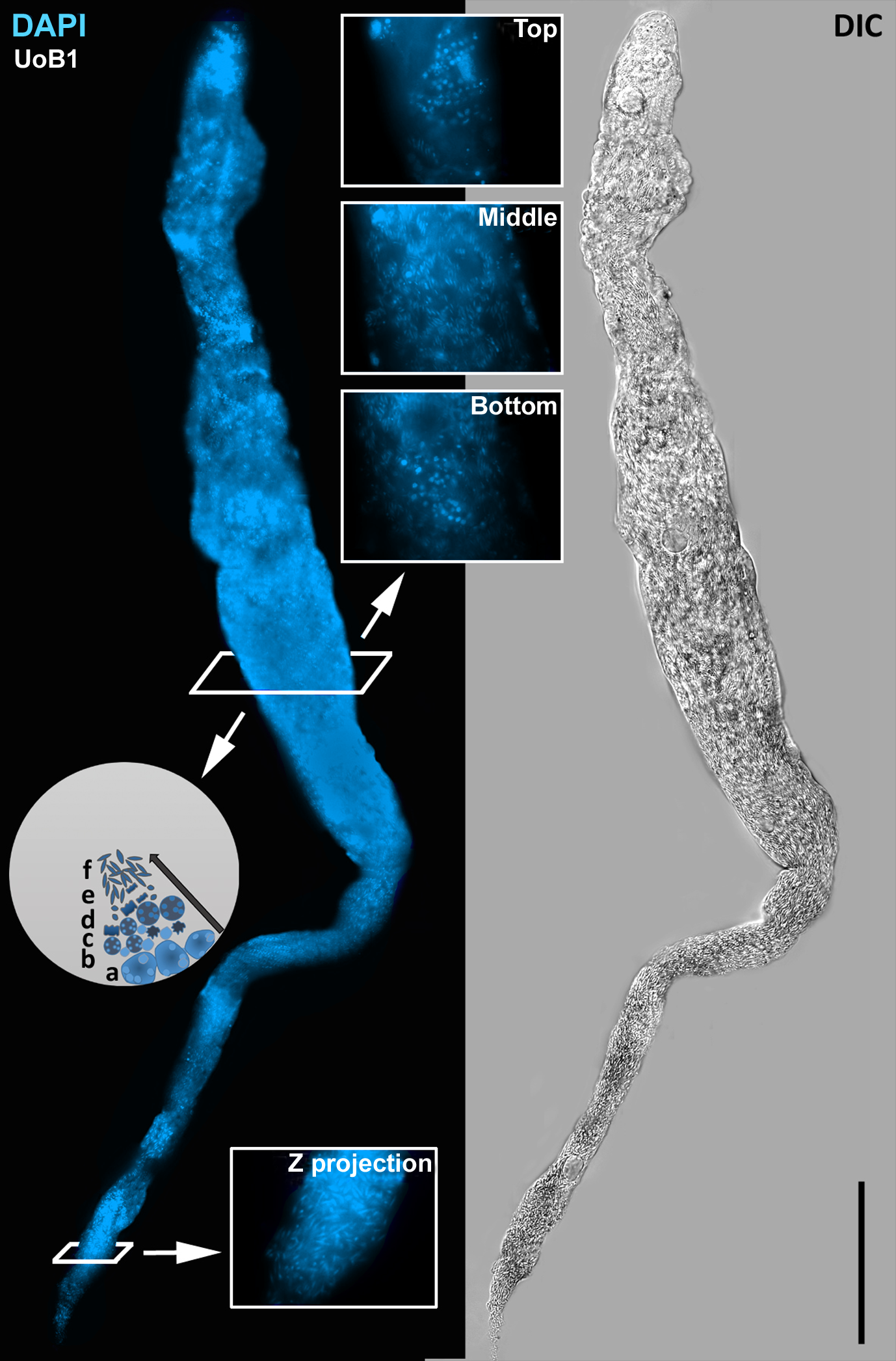

Supplement: Supplementary file 4 — High resolution image (TIF 1930 kb) [file 412_2015_558_MOESM2_ESM.tif]

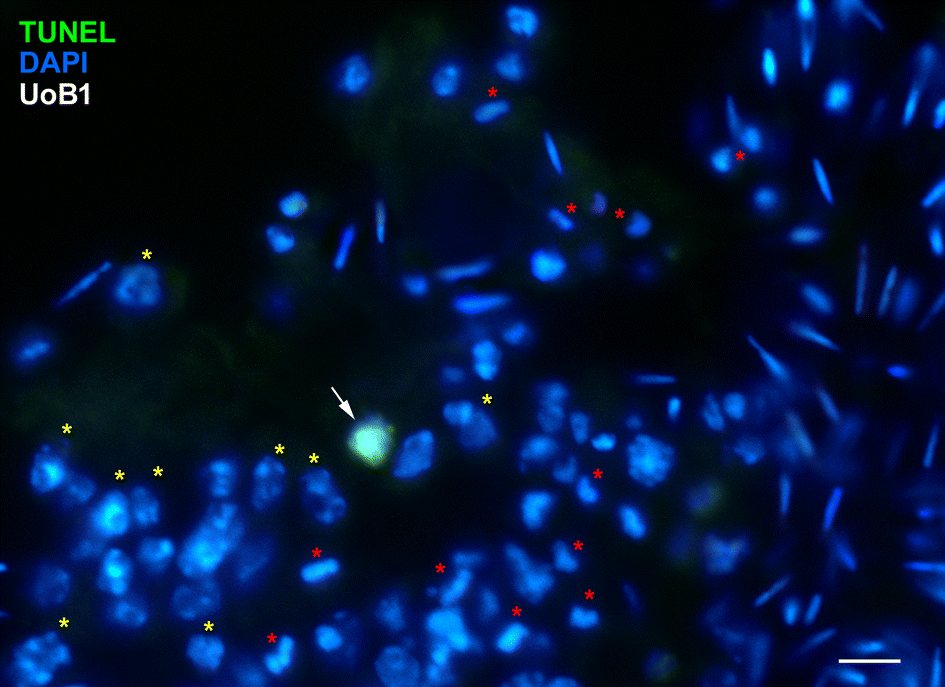

Supplement: Supplementary file 5 — TUNEL assay in UoB1. Spread of UoB1 Spermatocytes. DNA counterstained with DAPI (blue) and TdT-mediated dUTP-fluorescein nick end-labelling detecting DNA fragmentation-associated apoptosis of spermatocytes (green). The field shows several spermatocytes in prophase I (some of them indicated by a yellow star), and also dividing spermatocytes (some of them indicated by a red star). Only one spermatocyte in prophase I is detected as apoptotic in this field (white arrow). Scale bar corresponds to 10 μm. (GIF 388 kb) [file 412_2015_558_Fig12_ESM.gif]

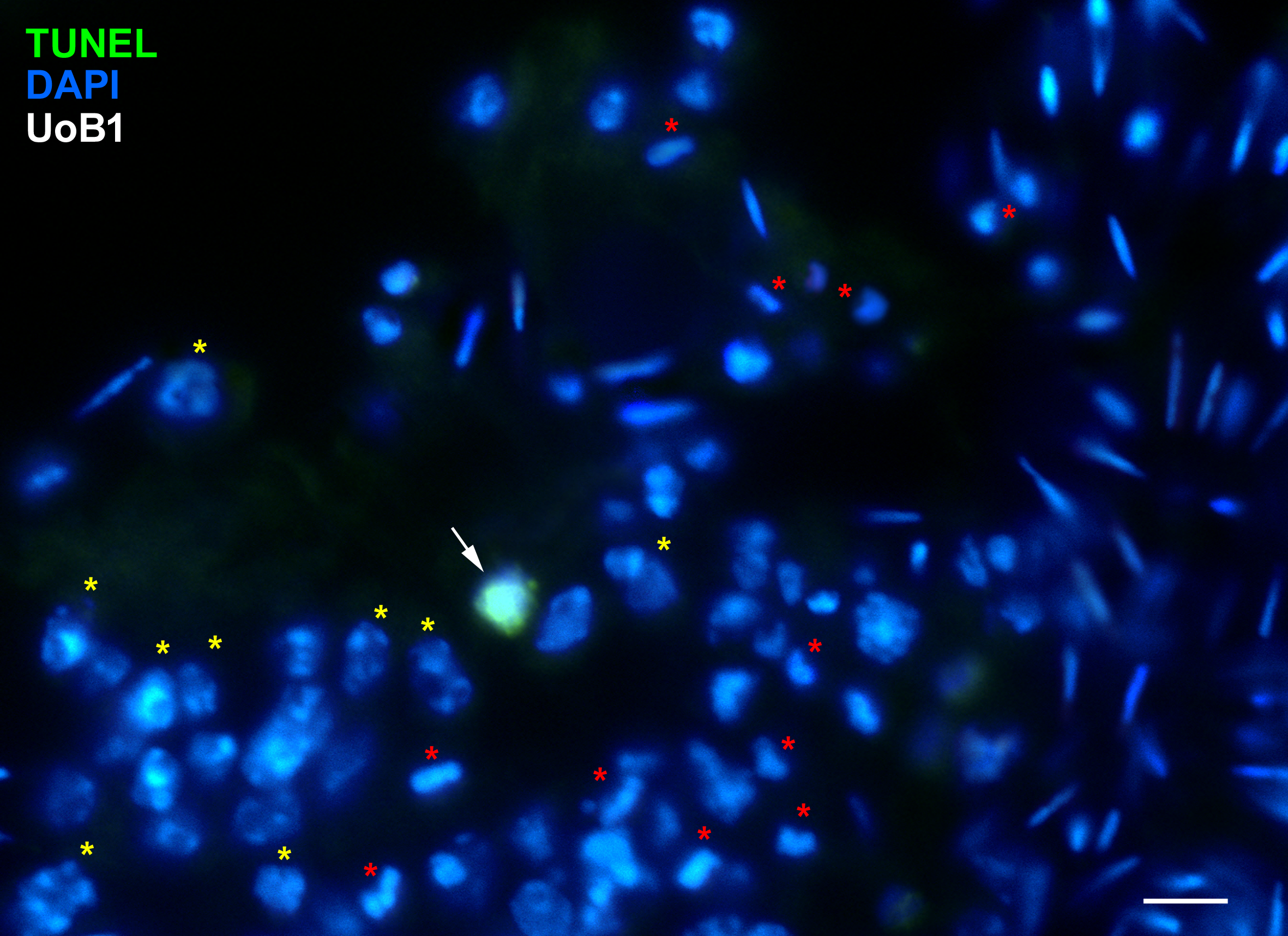

Supplement: Supplementary file 6 — High resolution image (TIF 11891 kb) [file 412_2015_558_MOESM3_ESM.tif]
