# Supplementary material for: Male meiosis in Crustacea: synapsis, recombination, epigenetics and fertility in Daphnia magna
Source: Chromosoma. 2015 Dec 21;125(4):769–87. doi: 10.1007/s00412-015-0558-1 (PMC5023733; doi:10.1007/s00412-015-0558-1)
Supplement: Supplementary file 7 — Detailed information of the study of the incidence of chromosomal aberrations in Daphnia magna line UoB1. (DOCX 18 kb) [file 412_2015_558_MOESM4_ESM.docx]

**Supplementary Table 1: Detailed information of the incidence of chromosomal aberrations in *Daphnia magna* line UoB1*.***

| **UoB1** | | | | | | |
| --- | --- | --- | --- | --- | --- | --- |
| **Metaphase I** | | | | | | |
| Individual | Number of cells analysed | Normal cells | Cells with univalents or misaligned bivalents | Percentage of cells with chromosomal aberrations | Total number of cells analysed | Average of cells with chromosomal aberrations among the five individuals |
| Individual 1 | 41 | 37 | 4 | 9.8% | 158 | 8,8% |
| Individual 2 | 28 | 26 | 2 | 7.1% |  |  |
| Individual 3 | 35 | 32 | 3 | 8.6% |  |  |
| Individual 4 | 23 | 21 | 2 | 8.7% |  |  |
| Individual 5 | 31 | 28 | 3 | 9.7% |  |  |
| **Telophase I** | | | | | | |
|  | Number of cells analysed | Normal cells | Cells with delayed chromosomes or chromatin bridges | Percentage of cells with chromosomal aberrations | Total number of cells analysed | Average of cells with chromosomal aberrations among the five individuals |
| Individual 1 | 30 | 28 | 2 | 6.7% | 143 | 6,2% |
| Individual 2 | 20 | 19 | 1 | 5.0% |  |  |
| Individual 3 | 33 | 31 | 2 | 6.1% |  |  |
| Individual 4 | 30 | 28 | 2 | 6.7% |  |  |
| Individual 5 | 30 | 28 | 2 | 6.7% |  |  |
| **Metaphase II** | | | | | | |
|  | Number of cells analysed | Normal cells | Cells with misaligned chromosomes | Percentage of cells with chromosomal aberrations | Total number of cells analysed | Average of cells with chromosomal aberrations among the five individuals |
| Individual 1 | 30 | 26 | 4 | 13.3% | 137 | 12,4% |
| Individual 2 | 27 | 24 | 3 | 11.1% |  |  |
| Individual 3 | 25 | 22 | 3 | 12.0% |  |  |
| Individual 4 | 25 | 22 | 3 | 12.0% |  |  |
| Individual 5 | 30 | 26 | 4 | 13.3% |  |  |
| **Telophase II** | | | | | | |
|  | Number of cells analysed | 9 | Cells with delayed chromosomes or chromatin bridges | Percentage of cells with chromosomal aberrations | Total number of cells analysed | Average of cells with chromosomal aberrations among the five individuals |
| Individual 1 | 20 | 17 | 3 | 15.0% | 129 | 10,6% |
| Individual 2 | 33 | 31 | 2 | 6.1% |  |  |
| Individual 3 | 26 | 24 | 2 | 7.7% |  |  |
| Individual 4 | 25 | 22 | 3 | 12.0% |  |  |
| Individual 5 | 25 | 22 | 3 | 12.0% |  |  |
