# Supplementary material for: Male meiosis in Crustacea: synapsis, recombination, epigenetics and fertility in Daphnia magna
Source: Chromosoma. 2015 Dec 21;125(4):769–87. doi: 10.1007/s00412-015-0558-1 (PMC5023733; doi:10.1007/s00412-015-0558-1)
Supplement: Supplementary file 8 — Detailed information of the study of the incidence of chromosomal aberrations in Daphnia magna line Xinb1. (DOCX 18 kb) [file 412_2015_558_MOESM5_ESM.docx]

**Supplementary Table 2: Detailed information of the incidence of chromosomal aberrations in *Daphnia magna* line Xinb1.**

| **Xinb1** | | | | | | |
| --- | --- | --- | --- | --- | --- | --- |
| **Metaphase I** | | | | | | |
| Individual | Number of cells analysed | Normal cells | Cells with univalents or misaligned bivalents | Percentage of cells with chromosomal aberrations | Total number of cells analysed | Average of cells with chromosomal aberrations among the five individuals |
| Individual 1 | 23 | 12 | 11 | 47.8% | 143 | 42,9% |
| Individual 2 | 30 | 18 | 12 | 40.0% |  |  |
| Individual 3 | 30 | 15 | 15 | 50.0% |  |  |
| Individual 4 | 29 | 18 | 11 | 37.9% |  |  |
| Individual 5 | 31 | 19 | 12 | 38.7% |  |  |
| **Telophase I** | | | | | | |
|  | Number of cells analysed | Normal cells | Cells with delayed chromosomes or chromatin bridges | Percentage of cells with chromosomal aberrations | Total number of cells analysed | Average of cells with chromosomal aberrations among the five individuals |
| Individual 1 | 16 | 10 | 6 | 37.1% | 117 | 52,5% |
| Individual 2 | 18 | 9 | 9 | 50.0% |  |  |
| Individual 3 | 24 | 7 | 17 | 70.8% |  |  |
| Individual 4 | 29 | 18 | 11 | 37.9% |  |  |
| Individual 5 | 30 | 10 | 20 | 66.7% |  |  |
| **Metaphase II** | | | | | | |
|  | Number of cells analysed | Normal cells | Cells with misaligned chromosomes | Percentage of cells with chromosomal aberrations | Total number of cells analysed | Average of cells with chromosomal aberrations among the five individuals |
| Individual 1 | 23 | 12 | 11 | 47,8% | 122 | 49,1% |
| Individual 2 | 18 | 9 | 9 | 50,0% |  |  |
| Individual 3 | 23 | 12 | 11 | 47,8% |  |  |
| Individual 4 | 28 | 14 | 14 | 50,0% |  |  |
| Individual 5 | 30 | 15 | 15 | 50,0% |  |  |
| **Telophase II** | | | | | | |
|  | Number of cells analysed | Normal cells | Cells with delayed chromosomes or chromatin bridges | Percentage of cells with chromosomal aberrations | Total number of cells analysed | Average of cells with chromosomal aberrations among the five individuals |
| Individual 1 | 25 | 13 | 12 | 48.0% | 130 | 41,6% |
| Individual 2 | 30 | 19 | 11 | 36.7% |  |  |
| Individual 3 | 30 | 18 | 12 | 40.0% |  |  |
| Individual 4 | 24 | 12 | 12 | 50.0% |  |  |
| Individual 5 | 21 | 14 | 7 | 33.3% |  |  |
