# Supplementary material for: Male meiosis in Crustacea: synapsis, recombination, epigenetics and fertility in Daphnia magna
Source: Chromosoma. 2015 Dec 21;125(4):769–87. doi: 10.1007/s00412-015-0558-1 (PMC5023733; doi:10.1007/s00412-015-0558-1)
Supplement: Supplementary file 9 — Detailed information of the study of the incidence of chromosomal aberrations in Daphnia magna line Xinb3. (DOCX 20 kb) [file 412_2015_558_MOESM6_ESM.docx]

**Supplementary Table 3: Detailed information of the incidence of chromosomal aberrations in *Daphnia magna* line Xinb3*.***

| **Xinb3** | | | | | | |
| --- | --- | --- | --- | --- | --- | --- |
| **Metaphase I** | | | | | | |
| Individual | Number of cells analysed | Normal cells | Cells with univalents or misaligned bivalents | Percentage of cells with chromosomal aberrations | Total number of cells analysed | Average of cells with chromosomal aberrations among the five individuals |
| Individual 1 | 30 | 10 | 20 | 66,7% | 143 | 66,6% |
| Individual 2 | 30 | 8 | 22 | 73,3% |  |  |
| Individual 3 | 25 | 8 | 17 | 68,0% |  |  |
| Individual 4 | 30 | 14 | 16 | 53,3% |  |  |
| Individual 5 | 28 | 8 | 20 | 71,4% |  |  |
| **Telophase I** | | | | | | |
|  | Number of cells analysed | Normal cells | Cells with delayed chromosomes or chromatin bridges | Percentage of cells with chromosomal aberrations | Total number of cells analysed | Average of cells with chromosomal aberrations among the five individuals |
| Individual 1 | 25 | 7 | 18 | 72,0% | 125 | 68,8% |
| Individual 2 | 26 | 6 | 20 | 76,9% |  |  |
| Individual 3 | 28 | 7 | 21 | 75,0% |  |  |
| Individual 4 | 20 | 6 | 14 | 70,0% |  |  |
| Individual 5 | 26 | 13 | 13 | 50,0% |  |  |
| **Metaphase II** | | | | | | |
|  | Number of cells analysed | Normal cells | Cells with misaligned chromosomes | Percentage of cells with chromosomal aberrations | Total number of cells analysed | Average of cells with chromosomal aberrations among the five individuals |
| Individual 1 | 29 | 15 | 14 | 48.3% | 142 | 59,4% |
| Individual 2 | 29 | 9 | 20 | 69.0% |  |  |
| Individual 3 | 29 | 14 | 15 | 51.7% |  |  |
| Individual 4 | 30 | 12 | 18 | 60.0% |  |  |
| Individual 5 | 25 | 8 | 17 | 68.0% |  |  |
| **Telophase II** | | | | | | |
|  | Number of cells analysed | Normal cells | Cells with delayed chromosomes or chromatin bridges | Percentage of cells with chromosomal aberrations | Total number of cells analysed | Average of cells with chromosomal aberrations among the five individuals |
| Individual 1 | 24 | 7 | 17 | 70.8% | 117 | 62,3% |
| Individual 2 | 28 | 11 | 17 | 60.7% |  |  |
| Individual 3 | 30 | 14 | 16 | 53.3% |  |  |
| Individual 4 | 15 | 5 | 10 | 66.7% |  |  |
| Individual 5 | 20 | 8 | 12 | 60.0% |  |  |

**Supplementary Table 4: Detailed information of the sperm quality analysis in *Daphnia magna* lines UoB1, Xinb1 and Xinb3.**

| **UoB1** | | | | | | |
| --- | --- | --- | --- | --- | --- | --- |
| Individual | Number of spermatozoa analysed | Spermatozoa with undamaged DNA | Spermatozoa with damaged DNA | Percentage of Spermatozoa with damaged DNA | Total number of Spermatozoa analysed | Average of spermatozoa with undamaged DNA |
| Individual 1 | 448 | 413 | 35 | 7,8% | 1771 | 6,7% |
| Individual 2 | 310 | 290 | 20 | 6,5% |  |  |
| Individual 3 | 331 | 312 | 19 | 5,7% |  |  |
| Individual 4 | 312 | 287 | 25 | 8% |  |  |
| Individual 5 | 370 | 349 | 21 | 5,67% |  |  |
| **Xinb1** | | | | | | |
|  | Number of spermatozoa analysed | Spermatozoa with undamaged DNA | Spermatozoa with damaged DNA | Percentage of Spermatozoa with damaged DNA | Total number of Spermatozoa analysed | Average of spermatozoa with undamaged DNA |
| Individual 1 | 432 | 383 | 49 | 11,3% | 2076 | 11,4% |
| Individual 2 | 514 | 441 | 73 | 14,2% |  |  |
| Individual 3 | 312 | 343 | 31 | 9,9% |  |  |
| Individual 4 | 335 | 302 | 33 | 9,9% |  |  |
| Individual 5 | 483 | 430 | 53 | 11,6% |  |  |
| **Xinb3** | | | | | | |
|  | Number of spermatozoa analysed | Spermatozoa with undamaged DNA | Spermatozoa with damaged DNA | Percentage of Spermatozoa with damaged DNA | Total number of Spermatozoa analysed | Average of spermatozoa with undamaged DNA |
| Individual 1 | 319 | 233 | 82 | 25,7% | 1478 | 25,6% |
| Individual 2 | 371 | 303 | 68 | 18,5% |  |  |
| Individual 3 | 314 | 212 | 102 | 32,5% |  |  |
| Individual 4 | 217 | 157 | 60 | 27,6% |  |  |
| Individual 5 | 257 | 212 | 42 | 17,5% |  |  |
