# Supplementary material for: Male meiosis in Crustacea: synapsis, recombination, epigenetics and fertility in Daphnia magna
Source: Chromosoma. 2015 Dec 21;125(4):769–87. doi: 10.1007/s00412-015-0558-1 (PMC5023733; doi:10.1007/s00412-015-0558-1)
Supplement: Supplementary file 13 — Spermatogonias are the primordial cells, which divide through mitosis to maintain a constant backup of cells. Their nuclei are big and possess numerous chromocentres that are clearly observed as hyperchromatic areas with DAPI (Fig. 1c). As chromatin condensation takes place, chromosomes begin to congress into the metaphase plate in spermatogonial prometaphase (Fig. 1d). When chromosomes are fully condensed and aligned, we observe spermatogonial metaphases in polar (Fig. 1e) or lateral (Fig. 1f) views. These spermatogonial metaphases are undergoing mitotic division, and therefore have 20 aligned chromosomes in D. magna. Identical sister chromatids of each chromosome are segregated during spermatogonial anaphase (Fig. 1g). After completing mitosis, some of the spermatogonias enter meiosis. During the first meiotic division, the prophase I stage lasts the longest. We identify leptotene/zygotene stages as small nuclei with homogeneous and hypochromatic chromatin (Fig. 1h). In pachytene, the chromatin has already begun condensation and the chromocentres—corresponding to the heterocentromeric chromatin surrounding the centromeres—are visible (Fig. 1i). In diplotene, the nuclear size is increased and chromocentres are clearly distinguished (Fig. 1j). In diakinesis, nuclei significantly increase in volume (Fig. 1k). Chromatid condensation and bivalent alignment take place during prometaphase I (Fig. 1l). In metaphase I, ten bivalents are perfectly aligned in the metaphase plate although the highly condensed chromatin does not allow the identification of each single one (Fig. 1m). To corroborate the alignment of the bivalents, we immunodetected these cells with αTubulin and we clearly observe a bipolar spindle (supplementary video 1). Homologues segregate during early anaphase I (Fig. 1n) and begin migrating to the cell poles in late anaphase I (Fig. 1o). Chromatin decondensation begins in early telophase I when homologues reach opposite poles (Fig. 1p). In late telophase I, [file 412_2015_558_MOESM13_ESM.docx]

**Additional supplementary legend of Figure 1.**

***Spermatogonias*** are the primordial cells, which divide through mitosis to maintain a constant backup of cells. Their nuclei are big and possess numerous chromocentres that are clearly observed as hyperchromatic areas with DAPI (Fig. 1 C). As chromatin condensation takes place, chromosomes begin to congress into the metaphase plate in ***spermatogonial prometaphase*** (Fig. 1 D). When chromosomes are fully condensed and aligned, we observe ***spermatogonial metaphases*** in polar (Fig. 1 E) or lateral (Fig. 1 F) views. These spermatogonial metaphases are undergoing mitotic division, and therefore have 20 aligned chromosomes in *D. magna*. Identical sister chromatids of each chromosome are segregated during ***spermatogonial anaphase*** (Fig. 1 G). After completing mitosis, some of the spermatogonias enter meiosis. During the first meiotic division, the ***prophase I*** stage lasts the longest. We identify ***leptotene/zygotene*** stages as small nuclei with homogeneous and hypochromatic chromatin (Fig. 1 H). In ***pachytene*,** the chromatin has already begun condensation and the chromocentres –corresponding to the heterocentromeric chromatin surrounding the centromeres– are visible (Fig. 1 I). In ***diplotene***, the nuclear size is increased and chromocentres are clearly distinguished (Fig. 1 J). In ***diakinesis*,** nuclei significantly increase in volume (Fig. 1 K). Chromatid condensation and bivalent alignment take place during ***prometaphase I*** (Fig. 1 L). In ***metaphase I***, ten bivalents are perfectly aligned in the metaphase plate although the highly condensed chromatin does not allow the identification of each single one (Fig. 1 M). To corroborate the alignment of the bivalents, we immunodetected these cells with αTubulin and we clearly observe a bipolar spindle (supplementary video 1). Homologues segregate during early ***anaphase I*** (Fig. 1 N) and begin migrating to the cell poles in late anaphase I (Fig. 1 O). Chromatin decondensation begins in early ***telophase I*** when homologues reach opposite poles (Fig. 1 P). In late telophase I, chromosome segregation is complete and we assume that the nuclear envelope begins to reconstitute when we observe round nuclei in opposite poles (Fig. 1 Q). Using αTubulin, we observe the contractile ring of cytokinesis between both daughter cells, demonstrating that these cells are indeed at the telophase stage (supplementary video 2). Each of these cells will progress to ***interkinesis*** (Fig. 1 R), an intermediate stage between both meiotic divisions when no DNA replication occurs. During ***prophase II,*** chromatin begins to condense and the nuclear envelope is disintegrated at the stage’s conclusion (data not shown). Ten chromosomes congregate during ***prometaphase II*** (Fig. 1 S) and finally align into the metaphase II plate (Fig. 1 T). Sister chromatids will segregate to opposite poles during anaphase II (Fig. 1 U) and the nuclear envelope will be reconstituted concomitantly with chromatin decondensation in ***telophase II*** (Fig. 1 V). Each of the haploids cells obtained after completion of meiosis, called early spermatids (Fig. 1 W), would enter a maturation process called ***spermiogenesis***, where chromatin will be highly compacted and nuclei shape will change from round to elongate in the medium spermatids (Fig. 1 X). Nuclei will again decrease in size to form mature spermatozoa (Fig, 1 Y).
